# DRAGON: Determining Regulatory Associations using Graphical models on multi-Omic Networks

**DOI:** 10.1093/nar/gkac1157

**Published:** 2022-12-19

**Authors:** Katherine H Shutta, Deborah Weighill, Rebekka Burkholz, Marouen Ben Guebila, Dawn L DeMeo, Helena U Zacharias, John Quackenbush, Michael Altenbuchinger

**Affiliations:** Department of Biostatistics, Harvard T.H. Chan School of Public Health, Boston, MA, USA; Channing Division of Network Medicine, Brigham and Women’s Hospital, and Department of Medicine, Harvard Medical School, Boston, MA, USA; Department of Biostatistics, Harvard T.H. Chan School of Public Health, Boston, MA, USA; CISPA Helmholtz Center for Information Security, Saarbrücken, Germany; Department of Biostatistics, Harvard T.H. Chan School of Public Health, Boston, MA, USA; Channing Division of Network Medicine, Brigham and Women’s Hospital, and Department of Medicine, Harvard Medical School, Boston, MA, USA; Department of Internal Medicine I, University Medical Center Schleswig-Holstein, Campus Kiel, Kiel, Germany; Institute of Clinical Molecular Biology, Kiel University and University Medical Center Schleswig-Holstein, Campus Kiel, Kiel, Germany; Peter L. Reichertz Institute for Medical Informatics of TU Braunschweig and Hannover Medical School, Hannover, Germany; Department of Biostatistics, Harvard T.H. Chan School of Public Health, Boston, MA, USA; Channing Division of Network Medicine, Brigham and Women’s Hospital, and Department of Medicine, Harvard Medical School, Boston, MA, USA; Department of Biostatistics, Harvard T.H. Chan School of Public Health, Boston, MA, USA; Department of Medical Bioinformatics, University Medical Center Göttingen, Göttingen, Germany

## Abstract

The increasing quantity of multi-omic data, such as methylomic and transcriptomic profiles collected on the same specimen or even on the same cell, provides a unique opportunity to explore the complex interactions that define cell phenotype and govern cellular responses to perturbations. We propose a network approach based on Gaussian Graphical Models (GGMs) that facilitates the joint analysis of paired omics data. This method, called DRAGON (Determining Regulatory Associations using Graphical models on multi-Omic Networks), calibrates its parameters to achieve an optimal trade-off between the network’s complexity and estimation accuracy, while explicitly accounting for the characteristics of each of the assessed omics ‘layers.’ In simulation studies, we show that DRAGON adapts to edge density and feature size differences between omics layers, improving model inference and edge recovery compared to state-of-the-art methods. We further demonstrate in an analysis of joint transcriptome - methylome data from TCGA breast cancer specimens that DRAGON can identify key molecular mechanisms such as gene regulation via promoter methylation. In particular, we identify Transcription Factor AP-2 Beta (TFAP2B) as a potential multi-omic biomarker for basal-type breast cancer. DRAGON is available as open-source code in Python through the Network Zoo package (netZooPy v0.8; netzoo.github.io).

## INTRODUCTION

Many biological systems can be visualized using networks, where biologically relevant elements are represented as nodes and relationships between those elements are represented as edges. Examples include gene regulatory networks, which represent the regulation of genes by transcription factors, and protein–protein interaction networks, which capture physical interactions between proteins (1,2). Network models can be based on prior knowledge (3), inferred from data (4), or combinations thereof (5). Here, we focus on data-driven network inference from high-throughput multi-omic data. In this context, co-expression networks (6), which are based on a measure of correlation such as Pearson’s correlation, are often used to capture potential associations between biomolecules that may be coordinately altered in specific biological states. However, a major drawback of such networks is that they do not distinguish direct from indirect effects (7). For example, consider a situation where a transcription factor *A* regulates the expression of two genes, *B* and *C*. In this case, a correlation network will contain an edge between gene *B* and gene *C* because correlation indicates a relation between the two genes. However, that relationship is only a consequence of their mutual relationship with the transcription factor *A* and thus the observed edge in the correlation network represents an indirect association. The problem of such erroneous correlations was discussed by Pearson and Yule in the early twentieth century, where the term ‘spurious correlation’ was introduced to distinguish indirect from direct relationships; a historical review of this question has been summarized by Aldrich and colleagues (8).

Several approaches have attempted to address this issue (9–12), of which Gaussian graphical models (GGMs; also known as partial correlation networks) (11,12) are among the most widely used methods. In a GGM, edges represent partial correlations. Intuitively, the partial correlation between two variables can be considered as the correlation that takes into account the effect of all remaining variables in the data set. Thus, it can distinguish a direct relationship from one that is mediated by one or more other variables. GGMs outperform simple correlation networks (13) and were consistently among the best in comparison to other methods in finding meaningful associations (14).

A single type of omics data generally only provides part of the information necessary to distinguish between direct and incidental relationships. For example, we know that gene regulation is a process that involves multiple layers of control, including transcription factor binding, epigenetic regulation, and chromatin structure. However, many analyses incorporate only gene expression data and not other regulatory data. Incorporating data from multiple omics could help prevent possibly erroneous conclusions based on the concept of spurious correlations introduced above.

New technologies are making it possible to generate multiple layers of omics data from the same samples. For example, The Cancer Genome Atlas (TCGA) provides data on RNA expression, methylation levels, and copy number variations for many individual tumor samples. More recently, single cell multi-omic data have become available as it has become possible to assay different omics data types from individual cells; for example, Cao *et al.* measured RNA and chromatin accessibility in single cells (15). Such multifactorial data will allow us to better disentangle interactions between biological variables and distinguish genuine from spurious associations.

Most omics data are high dimensional, meaning that the number of measured variables *p* typically exceeds the number of samples *n* (or both are of the same order of magnitude), which presents challenges for network inference (7). Several remedies have been proposed based on regularization techniques from high-dimensional statistics (11,12,16,17). Multi-omic network inference is complicated by a number of factors including the larger numbers of variables, different numbers of variables for each omics layer, variable noise levels within and between layers, and different edge densities in each data type.

In this work, we propose DRAGON, a machine learning method to estimate GGMs using two omics layers simultaneously. DRAGON calibrates omic-specific hyper-parameters for each omic layer to achieve an optimal trade-off between model complexity and estimation accuracy while explicitly taking into account the unique characteristics of each omics layer.

We show in simulations that DRAGON adapts to differences in edge densities and feature sizes of the included omics layers. Finally, we use DRAGON to analyze joint transcriptome–methylome data from TCGA breast cancer specimens. The latter analysis shows that DRAGON can identify potential regulatory molecular mechanisms, such as the association between promoter methylation and gene expression. We further show that DRAGON can identify multi-omic biomarkers, as exemplified by the combination of promoter methylation and gene-expression of TFAP2B (Transcription Factor AP-2 Beta), which is strongly associated with the basal-like breast cancer subtype.

## MATERIALS AND METHODS

### The Gaussian Graphical Model

Let *X* be a *n* × *p* data matrix of *n* observations (samples) and *p* features (such as genes, methylated sites, or proteins). Assume that the observations $\mathbf {x}_1,\mathbf {x}_2,\ldots , \mathbf {x}_n \in \mathbb {R}^p$ are independent and identically distributed according to a multivariate normal, *N*(μ, Σ), where Σ is a positive definite covariance matrix. Further, Θ = (θ_*ij*_) = Σ^−1^ is the inverse covariance matrix (also called precision matrix) where vanishing entries $\theta_{ij} = 0$ correspond to conditional independencies between variables *i* and *j*. A Gaussian Graphical Model (GGM) is a conditional dependence graph in which nodes represent variables and edges connect conditionally dependent pairs of variables (18,19).

Let $\mathcal{S}_n=\frac{1}{n}\sum _{i=1}^n(\mathbf {x}_i-\hat{\mu })(\mathbf {x}_i-\hat{\mu })^T$ be the sample covariance matrix, where $\hat{\mu }=\frac{1}{n}\sum _{i=1}^n \mathbf {x}_i$ is the sample mean. Then, the corresponding log likelihood takes the form

(1) $$\begin{equation*} l({\Theta })= \frac{n}{2}\left(\log |{\Theta }|-\operatorname{Tr}\left(\mathcal{S}_n{\Theta }\right)\right). \end{equation*}$$

### Covariance shrinkage

The maximum likelihood estimate (MLE) of Equation (1) yields $\hat{\Theta } = \mathcal{S}_n^{-1}$. However, if the number of features *p* exceeds the number of independent observations *n*, then $\mathcal S_n$ is singular and cannot be inverted. Even if *p* is smaller than *n* but of the same order of magnitude, $\hat{\Theta }$ has a high variance. One way this issue is often addressed is by adding regularization terms to Equation (1), as for example proposed in (17). Another approach is that of Schäfer *et al.* (12), who bias the covariance matrix towards a target matrix that is typically full-rank. Such ‘covariance shrinkage’ is based on the biased estimator

(2) $$\begin{equation*} {\hat{\Theta }} = \left((1-\lambda ){S}+\lambda {T}\right)^{-1}\, , \end{equation*}$$

where $S = \frac{1}{n-1}\sum_{i=1}^n(\mathbf{x}_i - \hat{\mu})(\mathbf{x}_i - \hat{\mu})^T = \frac{n}{n-1}\mathcal{S}_n$ is the unbiased empirical covariance, λ ∈ [0, 1] is a regularization parameter that can be estimated using the Lemma of Ledoit and Wolf (20) and *T* is the target matrix. Here, different choices for *T* have been proposed, such as the identity, *T* = *I*_*p*_, and the diagonal of *S*, ${T}=\operatorname{diag}(s_{11},s_{22},\ldots ,s_{pp})$. The idea behind Equation (2) is to replace the unbiased empirical covariance *S* with a linear combination of *S* and the target matrix *T* representing conditional independence. Because *T* is full-rank, the singularity of *S* is mitigated in this sum. Consequently, a biased precision matrix estimator can be obtained from inverting the shrunken covariance matrix. Throughout this article, we use the target matrix ${T}=\operatorname{diag}(s_{11},s_{22},\ldots ,s_{pp})$ following the arguments in (12).

### DRAGON

#### Generalized covariance shrinkage

In DRAGON, we extend covariance shrinkage to account for two different omics layers by introducing layer-specific regularization terms. Let *X*^(1)^ be a *n* × *p*_1_ data matrix that represents the first omics layer and let *X*^(2)^ be a *n* × *p*_2_ data matrix for the second layer, where *p*_1_ and *p*_2_ are the number of variables from omics layers 1 and 2. We further assume paired data, meaning that measurements (rows) $\mathbf {x}_i^{(1)}$ and $\mathbf {x}_i^{(2)}$ correspond to the same sample *i* but differ in their features. We define the empirical sample covariances $S^{(k, l)}=\frac{1}{n-1}\sum _{i=1}^n(\mathbf {x}_i^{(k)}-{\hat{\mu }}^{(k)})(\mathbf {x}_i^{(l)}-{\hat{\mu }}^{(l)})^T$ with the empirical mean vector $\hat{\mu }^{(k)}=\frac{1}{n}\sum _{i=1}^n \mathbf {x}_i^{(k)}$ for *k*, *l* ∈ {1, 2}. Now, we can generalize the shrinkage estimator to

(3) $$\begin{eqnarray*} {\hat{\Theta }}&=&\Bigg (\begin{pmatrix}(1-\lambda _1) {S}^{(1,1)}&\sqrt{1-\lambda _1}\sqrt{1-\lambda _2} S^{(1,2)}\nonumber \\ \sqrt{1-\lambda _1}\sqrt{1-\lambda _2} S^{(2,1)}&(1-\lambda _2) S^{(2,2)} \end{pmatrix}\nonumber \\ &&+\begin{pmatrix}\lambda _1 T^{(1)}&0\\ 0&\lambda _2 T^{(2)}\end{pmatrix}\Bigg )^{-1}\, , \end{eqnarray*}$$

with λ_*k*_ ∈ [0, 1] and ${T}^{(k)} = \rm {diag}(s^{(k)}_{11}, s^{(k)}_{22}, \ldots , s^{(k)}_{p_k p_k})$, where ${S}^{(k,k)}=(s^{(k)}_{ij})$. For illustration purposes, we first consider the limit λ_2_ = 1. Here, Equation (3) becomes

(4) $$\begin{eqnarray*} {\hat{\Theta }}&=&\left(\begin{pmatrix}(1-\lambda _1) S^{(1,1)}&0\\ 0&0 \end{pmatrix}+\begin{pmatrix}\lambda _1 T^{(1)}&0\\ 0& T^{(2)}\end{pmatrix}\right)^{-1}\nonumber \\ &=&\begin{pmatrix}\Theta ^{(1,1)}&0\\ 0&\left( T^{(2)}\right)^{-1} \end{pmatrix}\, , \end{eqnarray*}$$

where Θ^(1, 1)^ = ((1 − λ_1_)*S*^(1, 1)^ + λ_1_*T*^(1)^)^−1^ is the shrinkage estimator of the precision matrix using only the features with *k* = 1. Thus, if λ_2_ = 1, technology 1 decouples from technology 2.

Next, consider the limit λ_1_ = λ_2_ = λ. Then Equation (3) becomes

(5) $$\begin{eqnarray*} {\hat{\Theta }}&=&\left((1-\lambda )\begin{pmatrix}S^{(1,1)}& S^{(1,2)}\\ S^{(2,1)}& S^{(2,2)} \end{pmatrix}+\lambda \begin{pmatrix}T^{(1)}&0\\ 0& T^{(2)}\end{pmatrix}\right)^{-1}\nonumber \\ &=&\left((1-\lambda ) S+\lambda T\right)^{-1}\, . \end{eqnarray*}$$

Thus, we naively treat both omics layers as if they were generated using the same technology. These examples show that DRAGON naturally incorporates two limits that bound the optimal solution: (i) GGMs estimated for the two omics layers separately and (ii) a GGM treating both layers such as if they belong to the same layer.

#### Generalization of the lemma of Ledoit and Wolf

The penalty parameters λ_1_ and λ_2_ can be estimated using cross-validation or resampling; however, such approaches are computationally expensive. Alternatively, one can use an analytical estimate following the arguments of Ledoit & Wolf (20). There, the shrinkage parameter λ was derived by minimizing

(6) $$\begin{equation*} R=\operatorname{E}\!\left[|| {\hat{\Sigma }}-{\Sigma }||_F^2\right]\, \end{equation*}$$

with respect to λ, where ${\hat{\Sigma }}= (1-\lambda ){S} + \lambda {T}$ and Σ is the true, underlying covariance. This is possible since $\operatorname{bias}({S}) = 0$ makes Equation (6) independent of Σ.

Here, we extend this approach to the shrinkage formula (3) and estimate λ_1_ and λ_2_ by minimizing

(7) $$\begin{equation*} \operatorname{E}\!\left[|| {\hat{\Sigma }}-{\Sigma }||_F^2\right]=\sum _{k,l=1}^2\operatorname{E}\!\left[|| {\hat{\Sigma }}^{(k,l)}-{{\Sigma }}^{(k,l)}||_F^2\right] \end{equation*}$$

with respect to λ_1_ and λ_2_, where $|| \cdot ||_F$ is the Frobenius norm. Following the arguments in (12) (see Suppl. Section 1), we obtain

(8) $$\begin{eqnarray*} R&=&\operatorname{E}\!\left[|| {\hat{\Sigma }}-{\Sigma }||_F^2\right] \nonumber \\ &=&\rm {const.} + \lambda _1 T^{(1)}_{1}+ \lambda _2 T^{(2)}_{1}+ \lambda _1^2 T^{(1)}_{2}+ \lambda _2^2 T^{(2)}_{2} \nonumber \\ &&+ \lambda _1\lambda _2 T_{3}+ \sqrt{1-\lambda _1}\sqrt{1-\lambda _2}T_{4} \end{eqnarray*}$$

where the constant term is independent of λ_1_ and λ_2_, and

$$\begin{eqnarray*} T^{(k)}_{1}&=&-2\left(\sum _{i\ne j}\operatorname{var}(s^{(k)}_{ij})+\sum _{i, j}\operatorname{E}((s^{(1,2)}_{ij})^2)\right)\, ,\nonumber \\ T^{(k)}_{2}&=&\sum _{i\ne j} \operatorname{E}((s^{(k)}_{ij})^2)\, ,\nonumber \\ T_3&=&2\sum _{i, j}\operatorname{E}((s^{(1,2)}_{ij})^2)\, ,\nonumber \\ T_4&=&4\sum _{i, j}\left(\operatorname{var}(s^{(1,2)}_{ij})-\operatorname{E}((s^{(1,2)}_{ij})^2)\right)\, \nonumber \end{eqnarray*}$$

Equation (8) can be easily minimized with respect to λ_1_ ∈ [0, 1] and λ_2_ ∈ [0, 1], where the moments can be estimated following (12).

#### Hypotheses testing

An estimate for the partial correlation between variable *i* and *j* can be directly obtained from ${\hat{\Theta }}=( \hat{\theta }_{ij})$ by calculating

(9) $$\begin{equation*} \hat{\rho }_{ij} = -\frac{\hat{\theta }_{ij}}{\sqrt{\hat{\theta }_{ii}\hat{\theta }_{jj}}}\, . \end{equation*}$$

As a consequence of covariance shrinkage, the partial correlation matrix ${\hat{P}}=(\hat{\rho }_{ij})$ is also shrunken (21). Bernal et al. (2019) developed a null-model probability density that naturally accounts for this shrinkage effect (21):

(10) $$\begin{equation*} f^\lambda _{0}(\rho )=\frac{\left((1-\lambda )^2-\rho ^2\right)^{(\kappa -3)/2}}{\rm {Beta}(\frac{1}{2},\frac{\kappa -1}{2}) (1-\lambda )^{\kappa -2}}\, , \end{equation*}$$

where the parameter $\kappa$ is given by *n* − 1 − (*p* − 2) for *n* ≫ *p*, or can be fitted by MLE for the ill-posed case *p* > *n* or for *p* ≈ *n* (21). For an intuitive derivation of Equation (10) see (21). Let

(11) $$\begin{equation*} {\hat{\Theta }}=\begin{pmatrix}\hat{\Theta }^{(1,1)}&{\hat{\Theta }}^{(1,2)}\\ {\hat{\Theta }}^{(2,1)}&{\hat{\Theta }}^{(2,2)} \end{pmatrix}\quad {\rm and}\quad {\hat{P}}=\begin{pmatrix}\hat{P}^{(1,1)}&{\hat{P}}^{(1,2)}\\ {\hat{P}}^{(2,1)}&{\hat{P}}^{(2,2)} \end{pmatrix}\, , \end{equation*}$$

where ${\hat{\Theta }}^{(1,1)}$ (${\hat{P}}^{(1,1)}$) has dimension *p*_1_ × *p*_1_ and ${\hat{\Theta }}^{(2,2)}$ (${\hat{P}}^{(2,2)}$) dimension *p*_2_ × *p*_2_. Then, DRAGON assigns significance levels to partial correlations using the following steps:

- Simulate data under the null hypotheses (*H*_0_: ρ = 0) for given sample size *n*, and estimate corresponding partial correlations using DRAGON with λ_1_ and λ_2_ given from the original data.
- Fit $\kappa$ using MLE of Equation (10) for *P*^(1, 1)^, *P*^(1, 2)^ and *P*^(2, 2)^, separately.
- Use density Equation (10) with $\kappa$ determined in (ii) to assign significance levels to ${\hat{P}}^{(1,1)}$, ${\hat{P}}^{(1,2)}$, ${\hat{P}}^{(2,2)}$, respectively.
- Adjust significance levels in layer (1,1), (1,2) and (2,2) for multiple testing, separately, using the method of Benjamini and Hochberg (22). Note that if we control the false discovery rate (FDR) at level α in each layer, we also control the overall FDR at level α across all layers. However, to provide good estimates, *p*_1_ and *p*_2_ must be assumed to be sufficiently large.

For *n* ≫ *p*, $\kappa$ is given by $\kappa$ = *n* − 1 − (*p* − 2) and (i–ii) are not necessary.

### Performance comparisons

To benchmark the performance of DRAGON, we selected five methods for GGM estimation, each of which has distinct advantages and disadvantages (7), based on the requirement that they are available through a user-friendly software, provide estimates for *p*-values without computationally expensive resampling, and have been published in peer-reviewed journals.

- **GGM:** we implemented an omics-layer agnostic DRAGON model, which we simply denote as Gaussian Graphical Model (GGM) in the following. Note, in contrast to the GeneNet implementation described below (23), this approach uses the exact null distribution for shrunken partial correlations as suggested by (21).
- **GeneNet:** the R-package ‘GeneNet’ (23) uses covariance shrinkage and provides estimates for adjusted *p*-values (*q*-values) via an empirical Bayes approach (24). We used the standard settings for all comparisons.
- **B-NW-SL:** the bivariate nodewise scaled lasso was suggested by (25) and uses a regression approach to obtain asymptotically efficient estimates of the precision matrix under a sparseness condition relative to the sample size. We used the implementation provided in the ‘SILGGM’ R-package with standard settings.
- **D-S-NW-SL:** the de-sparsified nodewise scaled lasso (26) uses a modification of the nodewise lasso regression approach suggested by (16) with a de-sparsified estimator. We used the ‘SILGGM’ R-package with standard settings.
- **D-S-GL:** the de-sparsified graphical lasso was proposed by (27) and is based on a de-sparsified modification of the graphical lasso proposed by (17). We used the ‘SILGGM’ R-package with standard settings.

For all methods except GeneNet, which provides adjusted *p*-values (*q*-values), we adjusted *p*-values using the procedure of Benjamini and Hochberg (22).

## RESULTS AND DISCUSSION

### Simulation studies

We begin by briefly reviewing the concept of partial correlations and their relevance for multi-omics data analysis. Subsequently, we use four different simulation studies to show a comprehensive performance comparison between DRAGON and competing methods for GGM estimation.

**Figure 1.**
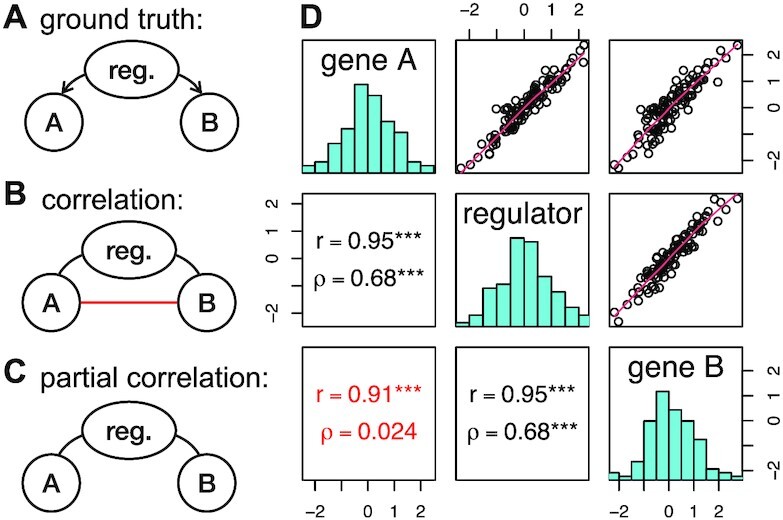
Partial correlation versus Pearson’s correlation on artificial data. The left figure shows from top to bottom: (**A**) the ground truth (directed), (**B**) the inferred correlation network (undirected), and (**C**) the inferred partial correlation network (undirected). Direct relationships are shown in black. Pearson’s correlation erroneously infers a relationship between *A* and *B* (figure **B**, red edge). Partial correlation correctly removes this relationship (figure **C**). The right figure (figure **D**) shows the corresponding data as scatterplots (upper half of the matrix) and histograms (diagonal). Corresponding Pearson’s correlation, *r*, and partial correlations, ρ, are given in the lower triangular matrix. Significance levels *P* < 0.001 are marked by three asterisks.

Multiple layers of interacting regulatory processes are involved in determining a cell’s state. By considering molecular variables of a single omic layer, such as the transcriptome, we can miss relevant information which might lead to erroneous conclusions about causal associations. To illustrate this effect, consider a scenario where the transcription of two genes, *A* and *B*, is regulated via the same molecular (*regulator*) ‘variable’. The *regulator* might belong to another omics layer representing a different biological factor such as the chromatin state in the DNA region of *A* and *B*. We generated artificial data representing such a process for *n* = 100 observations (Supplementary Section 2, Figure 1D), assuming a linear relationship between the observed level of the *regulator* and that of gene *A*, and between the *regulator* and gene *B*. Figure 1 B shows the corresponding correlation network and Figure 1D shows the estimated Pearson correlations, *r*, showing a high correlation between *A* and *B* (in red, *r* ∼ 0.91 and *p* < 0.001). This correlation is statistically significant although gene *A* and gene *B* are not directly related – rather, they are only co-regulated by the *regulator*. In contrast, the partial correlation between *A* and *B* takes into account the effect of the *regulator*, resulting in a non-significant partial correlation of ρ ∼ 0.02 (Figure 1C, using standard partial correlation) and a partial correlation network that does not have an edge between *A* and *B* (Figure 1 C), better reflecting the ground truth (Figure 1A). Thus, if the *regulator* is included in the model, it is statistically possible to disentangle direct and indirect linear relationships.

In this section, we systematically analyze potential issues in estimating partial correlations between variables of different omics layers, and compare how different methods, including DRAGON, are affected by these issues. In our simulations, we considered both high-dimensional settings (many molecular variables) as well as models in which there are different probabilities for direct relationships among variables within and between different omics layers. Table 1 describes the simulation study design in detail.

**Table 1.** Edge densities and edge numbers for simulation studies *A*, *B*, *C* and *D*

(*k*, *l*)	(1,1)		(1,2)		(2,2)	
	η	Edges	η	Edges	η	Edges
*A*	0.05	248	0.05	2500	0.05	6238
*B*	0.05	248	0.05	2500	0.005	624
*C*	0.05	248	0.005	250	0.005	624
*D*	0.101	500	0.01	500	0.004	500

We performed four simulation studies, *A* to *D*, with fixed numbers of molecular variables, *p*_1_ = 100 and *p*_2_ = 500 for omics layers 1 and 2, respectively. Direct relationships can be simulated by appropriate choice of the entries of the precision matrices ${\Theta }^{(k,l)}$ which parameterize the multivariate Gaussian distribution. Different simulation runs correspond to different precision matrices, which were randomly generated as follows:

1. start with a *p*-dimensional identity matrix, where *p* = *p*_1_ + *p*_2_,
2. randomly replace a proportion η^(*k*, *l*)^ of zeros by values drawn from a uniform distribution ranging from −1 to 1,
3. replace the diagonal entries $\theta_{ii}$ by $\sum_j|\theta_{ij}|$ plus a small positive value (ε = 0.0001),
4. normalize the entries $\theta_{ij}$ of the precision matrix by $\sqrt{\theta _{ii}\theta _{jj}}$.

Each precision matrix was used to sample from a multivariate normal with mean vector μ = 0 and covariance ${\Theta }^{-1}$. Finally, we added a noise ε ∼ *N*(μ = 0, σ = 0.1) to each entry of the data matrix.

#### DRAGON adapts to the data by dynamically chosen penalty parameters and improves model inference

DRAGON uses an analytical estimate of the minimum of *R*, Equation (6). Here, we show that this estimate dynamically adapts to sample and feature size, and to edge densities η^(*k*, *l*)^ across a broad range of simulation settings.

##### Parameter recovery in Simulation A

**Figure 2.**
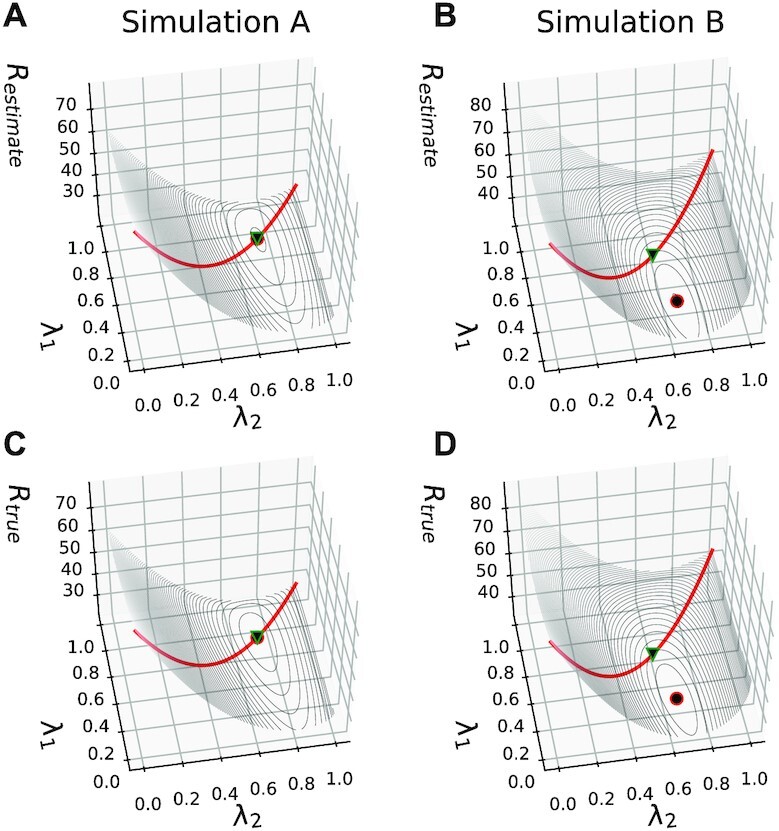
Parameter landscapes for DRAGON. Estimated and true *R* (upper and lower row) in dependency of λ_1_ and λ_2_ in simulation studies *A* (left column) and *B* (right column). Figures **A** and **B** show the estimated *R* for study *A* and *B*, respectively. Figure **C** and D show the corresponding ground truth. The red circles indicate the minima for each plot in the λ_1_–λ_2_ plane, and the green triangles give the minima on the diagonal *R* values shown in red, corresponding to the standard GGM.

In simulation study *A*, the two omics layers have equal edge densities η^(1, 1)^ = η^(1, 2)^ = η^(2, 2)^ = 0.05. Figure 2A and C shows *R* in dependency of λ_1_ and λ_2_ for the analytical estimate and the ground truth, respectively, for a fixed sample size of *n* = 5000. We observed that estimate and ground truth agree remarkably well, and that they provide almost identical estimates for the position of the minimum of the selected penalty parameters, $(\hat{\lambda }_1,\hat{\lambda }_2)$. The GGM estimated according to Equation (2) using the appended data *X* = [*X*^(1)^, *X*^(2)^] corresponds to the diagonal red lines, such that the two data layers are treated as they would belong to one layer and a single regularization parameter $\hat\lambda_1 = \hat\lambda_2 = \hat\lambda$ is estimated. We observed that DRAGON correctly estimated $(\hat{\lambda }_1,\hat{\lambda }_2)$ to be near to the diagonal for this simulation study. Supplementary Figures S1 and S2 confirm this finding for lower sample sizes, *n* = 500 and *n* = 1000, respectively.

##### Parameter recovery in simulation B

In Simulation B, we investigated the influence of the edge densities η^(*k*, *l*)^ on *R*. For this, we reduced the edge density η^(2, 2)^ to η^(2, 2)^ = 0.005. Again, we observed a remarkable agreement between estimated and true *R*, shown in Figure 2B and D, respectively, which is also the case for the estimated minima $(\hat{\lambda }_1,\hat{\lambda }_2)$. Note, $\hat{\lambda }_1$ now strongly differs from $\hat{\lambda }_2$. Since $\lambda_2 >\lambda_1$, the second omics data layer is stronger penalized than the first.

##### Parameter recovery in simulations C and D

We repeated this analysis for two further simulation studies, *C* and *D*, both of which have unbalanced edge densities with results shown in Supplementary Figures S3, S4, and S5 for *n* = 500, *n* = 1000, and *n* = 5000, respectively. Here, we also found that DRAGON correctly estimates *R* and that λ_1_ and λ_2_ are chosen by the algorithm accordingly to minimize *R*.

##### Model inference in simulations A–D

We next analyzed how the DRAGON regularization scheme, which augments covariance shrinkage by omics layer-specific regularization parameters, affects model inference. We repeated simulation studies *A* to *D* 20 times for different sample sizes *n* and evaluated the log-likelihood on *n* = 1000 test samples (Supplementary Figure S6). While we recognize that few omics studies have 1000 or more samples, single-cell experiments generally assay thousands of cells and large cohort studies are beginning to develop omics databases that include populations of this size or larger.

We found similar results on an absolute scale (Supplementary Figures S6a–d). However, results were clearly in favor of DRAGON when we evaluated the log-likelihood difference (Supplementary Figures S6e–h). This measure has the advantage that it removes variability due to different simulation runs. The green line corresponds to the median log-likelihood difference and the error bands to the $25\%$ and $75\%$ percentiles. Results were slightly in favor of the GGM for the balanced study *A*, but in simuations *B*, *C*, and *D*, DRAGON outperformed the standard GGM as indicated by positive values for the difference. The greatest improvements were seen for the unbalanced simulation *B*. We can also see that as the sample size *n* increases with the number of predictors *p* remaining fixed, DRAGON and GGM estimates coincide, as demonstrated by a vanishing log-likelihood difference.

#### DRAGON outperforms the state of the art with respect to edge recovery

Adjusted *p*-values (*q*-values) were assigned as described in the Materials and Methods section and were used to assess the edge-recovery performance in simulations *A* to *D* using receiver operating characteristic (ROC) curves for DRAGON, GGM, GeneNet, B-NW-SL, D-S-NW-SL and D-S-GL. Variables were standardized for performance comparisons.

**Figure 3.**
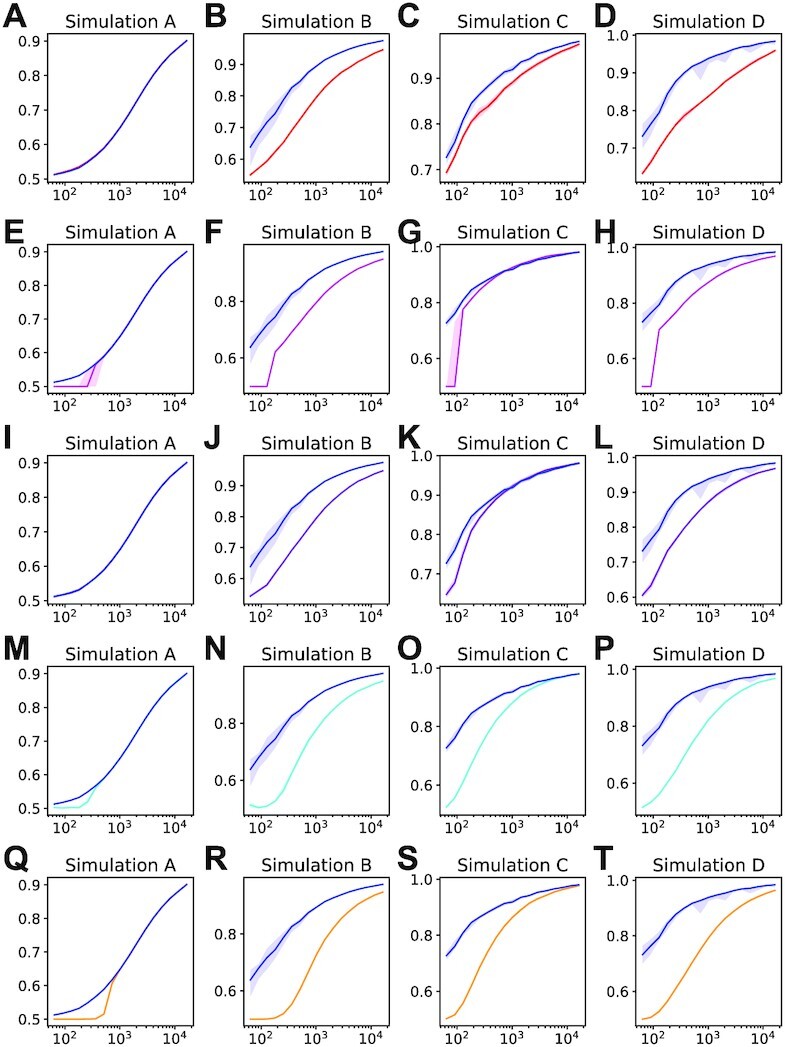
Area under the ROC curve (AUC) for edge-recovery performance versus sample size *n* of simulation studies *A* to *D* (columns). Each row compares DRAGON (blue) with GGM (red, **A–D**), GeneNet (magenta, **E–H**), B-NW-SL (purple, **I–L**), D-S-NW-SL (cyan, **M–P**) and D-S-GL (orange, **Q–T**), respectively. The lines correspond to the median AUC and the bands to the $25\%$ and $75\%$ percentiles of the distribution.

**Figure 4.**
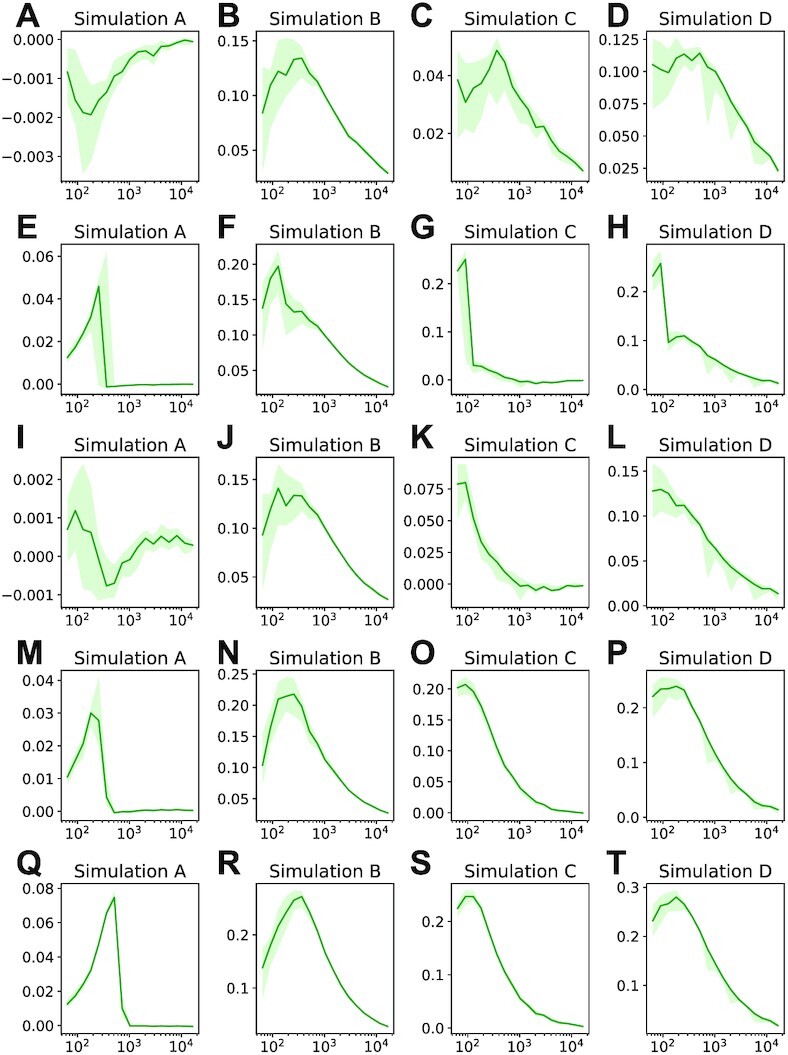
Area under the ROC curve (AUC) differences versus sample size *n* of simulation studies *A* to *D* (columns). Each row corresponds to the AUC difference DRAGON minus GGM (**A–D**), DRAGON minus GeneNet (Figure **E**–**H**), DRAGON minus B-NW-SL (**I–L**), DRAGON minus D-S-NW-SL (**M–P**), and DRAGON minus D-S-GL (**Q–T**), respectively. The green lines correspond to the median AUC and the bands to the $25\%$ and $75\%$ percentiles of the distribution.

We first compared the results of DRAGON and GGM inference methods. Figure 3A shows how the area under the ROC curve (AUC) depends on the number of training samples *n* for simulation study *A*. We saw almost identical performance for DRAGON (blue) and GGM (red) for all sample sizes in our simulation, a result consistent with our previous findings. For simulation studies *B*, *C* and *D*, the corresponding results are shown in Figure 3B–D and illustrate substantial improvements for DRAGON compared to GGM. For example, in simulation study *B*, DRAGON achieved an AUC for *n* ≈ 250 samples that is compatible to that of the GGM for *n* ≈ 1000 samples. This improved performance of DRAGON is further illustrated by Figure 4 A to D that shows the respective AUC differences; we found AUC improvements up to ∼0.13 (study *B*), ∼0.05 (study *C*) and ∼0.11 (study *D*). In the balanced simulation scenario *A* where we expect the GGM and DRAGON to have similar performance, the GGM negligibly outperformed DRAGON, as shown by small negative values in Figure 4A. As an alternative performance assessment, we used the average area under the precision-recall curve (AUC-PR) (Supplementary Figure S7). These results also show the performance advantages of DRAGON compared to GGM with AUC-PR differences up to ∼0.07, ∼0.03, ∼0.08, for studies *B* to *D*, respectively.

Analogous AUC analyses were performed comparing DRAGON with GeneNet, B-NW-SL, D-S-NW-SL and D-S-GL with results shown in Figures 3E–T and 4E–T. Corresponding AUC-PR curves and AUC-PR differences are shown in Supplementary Figures S7 and S8, respectively. For both AUC and AUC-PR, we found in simulated data sets *A*, *B* and *D* that DRAGON’s improved estimates are of the same size (for GeneNet and B-NW-SL) or substantially larger (for D-S-NW-SL and D-S-GL) than we found in comparing DRAGON to GGM. In simulation study *C*, we found comparable performance of GeneNet and DRAGON across a large range of sample sizes (Figures 3G and 4G) but that GeneNet produced an inflated FDR (Supplementary Figure S9g). FDRs and FDR differences for all methods are summarized in Supplementary Figures S9 and S10. Table 2 summarizes the minimum number of samples *n* (as well as upper bounds on *p*/*n*) for each of the simulation studies required to reach network confidence levels AUC >0.8. As can be seen, DRAGON reached confidence for substantially smaller *n* and larger *p*/*n* ratios than the other methods.

**Table 2.** Lower bounds on *n* and upper bounds on *p*/*n* necessary to achieve confidence in edge recovery defined via a median AUC>0.8 in simulation studies *A*, *B*, *C* and *D*. Best performance is indicated in boldface letters. The evaluated sample sizes were *n* ∈ {2^6^, ⌊2^6.5^⌋, 2^7^, …, ⌊2^13.5^⌋, 2^14^}

Simulation	*A*		*B*		*C*		*D*	
	*n*	*p*/*n*	*n*	*p*/*n*	*n*	*p*/*n*	*n*	*p*/*n*
DRAGON	4096	0.15	**362**	**1.66**	**128**	**4.69**	**181**	**3.31**
GGM	4096	0.15	1448	0.41	181	3.31	512	1.17
GeneNet	4096	0.15	1448	0.41	181	3.31	512	1.17
B-NW-SL	4096	0.15	1448	0.41	181	3.31	512	1.17
D-S-NW-SL	4096	0.15	1448	0.41	512	1.17	1024	0.59
D-S-GL	4096	0.15	2048	0.29	724	0.83	1448	0.41
								

We also verified that DRAGON correctly estimates *P*-values and false discovery rates (FDRs). First, we performed simulations under the null hypothesis of no partial correlation (ρ = 0) and verified that the *P*-value distributions are flat for different sets of the regularization parameters λ_1_ and λ_2_ (Supplementary Figures S12 and S13). The simulated data sets were motivated as follows: we recorded the λ_1_ and λ_2_ values for each simulation run in study *A* to *D*, which yielded the results shown in Supplementary Figure S12. We extracted the corresponding parameter sets at *n* = 256, *n* = 1024 and *n* = 4096 samples, which correspond respectively to a highly regularized estimate, a moderately regularized one, and one with low regularization. This resulted, in total, in 12 pairs (λ_1_, λ_2_) with the associated *P*-value distributions under the null hypothesis that are shown in Supplementary Figure S13; as desired, these distributions are flat. Further, we recorded the FDRs corresponding to studies *A* to *D* (Supplementary Figures S9a to d and S11) for the empirical estimate of κ and the theoretical value κ = *n* − 1 − (*p* − 2), respectively. As expected, the observed FDR for DRAGON approaches 0.05 as the sample size increases. Analogous plots for the comparison methods can be found in Supplementary Figure S9e–t. DRAGON generally outperforms the other methods, some of which show inflated FDRs indicating an overly liberal method (GeneNet) or deflated FDRs indicating an overly conservative method (D-S-NW-SL and D-S-GL).

### Integrative DRAGON analysis of transcriptome and methylome in TCGA breast cancer specimens

Epigenetic aberrations such as DNA methylation are associated with altered patterns of gene regulation during the development and progression of complex diseases such as cancer (28). Given the performance advantages of DRAGON over other partial correlation methods, here we present an illustrative application of DRAGON to integrate promoter methylation and gene expression data in 765 breast cancer specimens from The Cancer Genome Atlas (TCGA) (29). We began with a list of 1639 human transcription factors (TFs) (30), of which 1557 had annotated promoter methylation data meeting quality control measures in TCGA, 1311 had gene expression data, and 1280 had both. Below, we outline the analysis of these data; further details regarding the sample population, data preprocessing, model inference, community detection and Reactome pathway enrichment analysis are summarized in Supplementary Sections 4–8, where a summary of baseline characteristics is given in Suppl. Table S1, the distribution of promoter methylation is shown in Supplementary Figure S14, and the distribution of methylation in relation to CpG islands is shown in Supplementary Figure S15.

#### Data acquisition, processing and estimated DRAGON model

The GenomicDataCommons (31) R package, version 1.20.1, was used to download TCGA breast cancer data (Project ID TCGA-BRCA; dbGaP Study Accession phs000178) from the Genomic Data Commons. Methylation levels from the Illumina 450k array were processed from raw data into beta values with the TCGA Methylation Array Harmonization Workflow (https://docs.gdc.cancer.gov/Data/Bioinformatics_Pipelines/Methylation_Pipeline/, Accessed: 2022-05-25), which uses SeSAMe (32) for signal detection and quality control. Methylation was summarized at the gene level by averaging beta values for probes in the promoter region of each gene of interest and methylation data were transformed to approximate normality with the nonparanormal transformation (33). RNA-seq data were processed according to the TCGA RNA-Seq Alignment Workflow (https://docs.gdc.cancer.gov/Data/Bioinformatics_Pipelines/Expression_mRNA_Pipeline/, Accessed: 2022-05-25) to produce gene expression levels; this pipeline uses the Spliced Transcripts Alignment to a Reference (STAR) (34) algorithm to align reads and generate counts which were reported as transcripts per million (TPM), among other measures.

**Figure 5.**
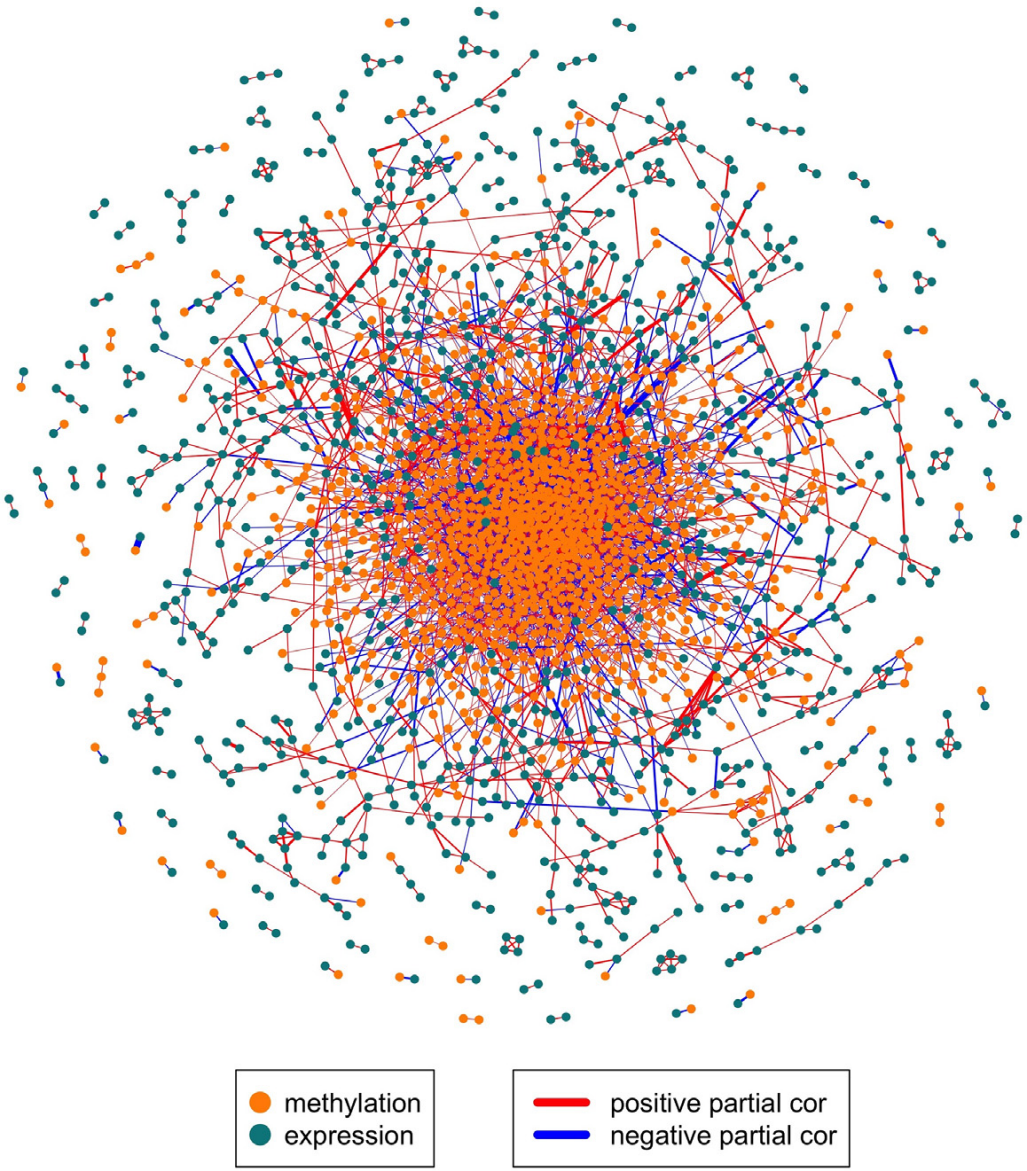
DRAGON multiomic network on TCGA methylation and gene expression data from 765 women with breast cancer.

Methylation and expression data for the transcription factors for this breast cancer data set were loaded into DRAGON and a partial correlation network was calculated. A thresholded version of the DRAGON-estimated network consisting of all edges with FDR <0.005 and all nodes with degree >0 is shown in Figure 5. The network contains 3631 edges on 2106 nodes. 1168 of the nodes represent methylation (75% of the methylation variables) and 938 represent gene expression (72% of the gene expression variables).

#### DRAGON discovers meaningful promoter methylation-gene expression relationships

DNA methylation can regulate gene expression by blocking TF binding or by altering the binding of other regulatory proteins (35). In cancer, hypermethylation of tumor suppressor gene promoters can lead to their inactivation by blocking transcription factors and inhibiting the recruitment of the transcriptional machinery (36). On the other hand, DNA methylation results from the actions of proteins (methylases and demethylases) whose levels in turn result from changes in the expression of genes encoding these proteins. DRAGON provides a means to study both changes in gene expression resulting from methylation and the activation of methylation through changes in gene expression.

Because of the complex, correlated nature of methylation and gene expression, a multi-omic correlation network based on these data may be too noisy to identify meaningful methylation-expression relationships. To illustrate this, we created a Pearson correlation network on the methylation-gene expression data with 824,599 significant methylation-gene expression edges based on the criteria FDR <0.05. Notably, of these edges, only 861 (0.1%) were edges between the expression of a gene and the methylation of its promoter – the associations we expect functionally to be the most significant (175 (20%) positive and 686 (80%) negative edges). In contrast, when using DRAGON to estimate the partial correlation network on the same data, the result was a much sparser network consisting of 769 significant methylation-gene expression edges (FDR <0.05). Of these edges, 333 (43%) were associations between expression of a gene and methylation of its promoter (26 (8%) positive and 307 (92%) negative partial correlations). The high proportion of negative partial correlations is an important ‘sanity check’ on the performance of DRAGON, as promoter methylation suppresses transcription for most genes (36). To further illustrate the discriminatory power of partial correlations relative to simple correlations, we examined the 769 most significant Pearson correlation edges between gene expression and methylation sites (corresponding to the number of gene expression-methylation edges in the DRAGON network) and found only 33 (4%) to be associations between expression of a gene and methylation of its promoter. Note that neither the Pearson correlation-based analysis nor the DRAGON analysis use any prior knowledge about methylation site - gene correspondence.

**Figure 6.**
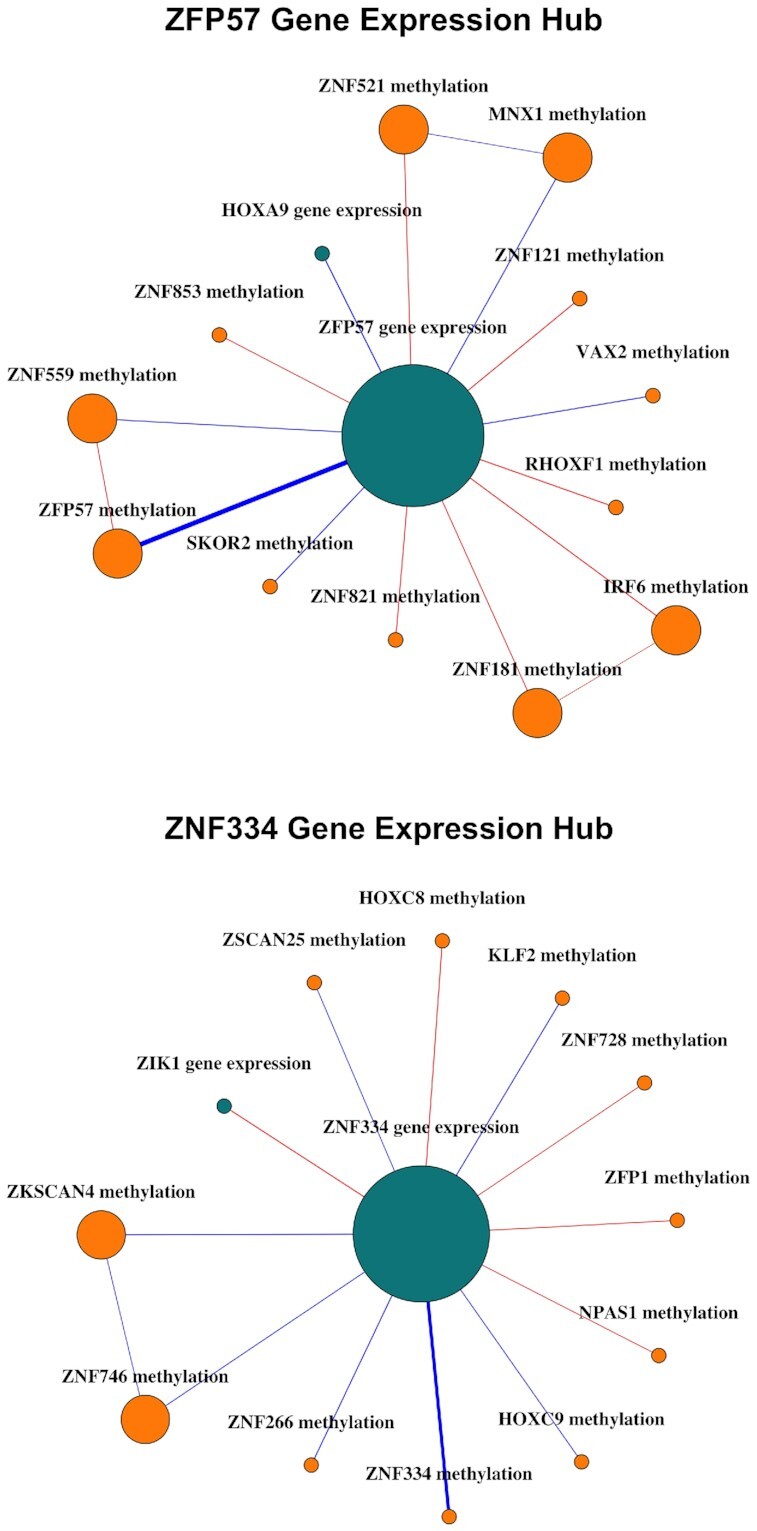
Neighborhoods of ZFP57 and ZNF334, two of the transcription factor gene expression nodes that serve as hubs in the DRAGON breast cancer network. Turquoise nodes represent gene expression and orange nodes represent promoter methylation. Red edges indicate positive partial correlations; blue, negative. Edge width is proportional to the strength of partial correlation. Larger nodes indicate higher node degrees. Edges with FDR <0.05 are shown.

Returning to the DRAGON network, we ranked gene expression nodes according to the number of significant edges to methylation nodes they possessed (Table 3). The top-ranking gene, with 12 edges to methylation nodes (7 positive and 5 negative partial correlations), was ZFP57 (Figure 6) which is a zinc finger transcription factor containing a KRAB domain and which may play a negative regulatory role (37). The strongest gene expression - promoter methylation edge was observed between ZFP57 and methylation of its own promoter (FDR < 10^−100^, ρ = −0.25), followed by methylation of the SKOR2 promoter (FDR < 10^−3^, ρ = −0.06), and methylation of the ZNF121 promoter (FDR < 10^−3^, ρ = 0.06). A comprehensive list of edges is shown in Table 3. ZFP57 is known to contribute in maintaining the methylation memory of parental origin (38). It controls DNA methylation during the earliest multicellular developmental stages at multiple imprinting control regions (39,40), which is in line with its multiple related methylation sites suggested by DRAGON. Most importantly, ZFP57 has been shown to suppress proliferation of breast cancer cells through down-regulation of the MEST-mediated Wnt/β-catenin signalling pathway (41).

**Table 3.** Summary of the 4 gene-expression variables with most edges to methylation variables ordered by rank. Methylation variables (last column) are ordered by partial correlations (from high to low absolute values), where the +/− signs in brackets give the signs of the estimated partial correlations

rank	TF RNA	# GE-methyl. edges	Methylation variables
1	ZFP57	12	ZFP57(−), SKOR2(−), ZNF121(+), IRF6(+), RHOXF1(+), MNX1(−), VAX2(−),
			ZNF821(+), ZNF181(+), ZNF559(−), ZNF521(+), ZNF853(+)
2	ZNF334	11	ZNF334(−), ZNF266(−), KLF2(−), ZKSCAN4(−), HOXC8(+), ZNF746(−),
			HOXC9(−), ZFP1(+), ZNF728(+), ZSCAN25(−), NPAS1(+)
3	NR6A1	10	FOXO6(+), IKZF3(+), JRK(−), NR5A1(+), NFIC(+), ZNF654(−), FOXR1(−),
			ETV2(+), ELK3(+), FOXP1(−)
4	MYRFL	10	MYRFL(−), ZFP69B(−), AHR(−), HOXB7(+), ZNF467(−), THAP12(+), ZNF251(−), MTF1(+), ARID5A(+), RORB(+)

The second TF was ZNF334, another zinc finger protein transcription factor, which had 11 edges to methylation variables (4 positive, 7 negative; Figure 6). Again, the strongest edge was observed to its own methylation site (FDR <10^−34^, ρ = −0.175), followed by ZNF266 (FDR =3.6 × 10^−4^, ρ = −0.058) and KLF2 (FDR =7.6 × 10^−4^, ρ = −0.056). ZNF334 was recently identified as tumor suppressor of triple-negative breast cancer and higher ZNF334 expression was shown to be associated with better survival outcomes (42). DRAGON suggests that this suppression may be due to hypermethylation of its promoter region. A similar pattern has been observed in some other cancers, including hepatocellular carcinoma (43).

The third and fourth top hits were the transcription factors NR6A1 (also known as GCNF) and MYRFL, both showing 10 edges to methylation variables (Supplementary Figure S16). Unlike the other transcription factors we identified, NR6A1 was not related to its own methylation site (FDR ∼1, ρ ∼ 0). Its strongest methylation-gene expression edges were observed to methylation sites attributed to the genes FOXO6 (FDR <1 × 10^−4^, ρ = 0.061) and IKZF3 (FDR <1 × 10^−4^, ρ = 0.061). FOXO6 is a transcription factor known to play multiple roles in breast cancer. Its downregulation is implicated in promotion of the epithelial–mesenchymal transition, in migration and proliferation of breast cancer cells, and in reduced cell resistance to the anti-cancer drug paclitaxel through the PI3K/Akt signaling pathway (44,45). IKZF3 is a member of the Ikaros family of zinc-finger proteins that has been shown to work with other transcription factors to regulate immune response in breast cancer (46) and its knockdown has been shown to dramatically increase breast cancer response to chimeric antigen receptor T-cell (CAR-T) therapy (47).

In *mus musculus*, NR6A1 has been shown to interact with DNMT3B (DNA (cytosine-5)-methyltransferase 3B) to induce promoter methylation of the genes Oct-3/4 (48). DNMT3B together with DNMT3A is essential for the *de novo* methylation in early development (49). In addition to its role as a transcription factor, NRA61 is an orphan nuclear receptor normally expressed in germ cells of gonads and highly expressed in triple-negative and ER+ HER2– breast cancer and so has been suggested as an ideal drug target (50). MYRFL follows the more typical pattern, having its strongest methylation-gene expression edge to its own methylation site (FDR <1 × 10^−7^, ρ = −0.073). MYRFL encodes a transcription factor that is required for central nervous system myelination and it has been identified as a member of a regulatory cluster of genes on chromosome 12 that has been associated with elevated risk of breast cancer (51).

Although this analysis does not fully account for either the complexities of breast cancer and its subtypes or for the interplay of regulatory mechanisms active in cells and limited exploration to the transcription factors themselves, it already paints a compelling picture of the interplay between epigenetic regulation through altered patterns of methylation in breast cancer and activation or repression of key regulatory proteins that control breast cancer risk, cell proliferation and response to various therapeutic interventions.

#### Community detection and enrichment analysis

To further analyze the complex structure of the estimated network, we clustered nodes using community detection on the DRAGON-estimated network thresholded at FDR <0.005. This threshold was based on inspection of the distribution of FDR-corrected *p*-values; Supplementary Figure S17. Community detection was performed using the cluster_fast_greedy algorithm as implemented in the igraph R package (52,53) (see Supplement). Using this algorithm, 169 communities were detected, 59 of which had at least 5 nodes.

To assess the enrichment of methylation-gene expression communities for functions potentially related to breast cancer, we performed an over-representation analysis (ORA) for Reactome gene sets (54) within each of the 59 communities with at least 5 nodes (for details of the ORA, see Supplement). The fora function of the fgsea R package was used to conduct the ORA (55). Reactome gene sets with at least 3 genes were considered (minSize = 3). For the analysis presented here, it should be noted that each transcription factor gene may appear twice, once based on its expression and once based on its methylation. Therefore, the universe of possible genes considered for the ORA (and used to set background expectations) is twice the size of the number of TFs included in the DRAGON model. We also performed an ORA assessing enrichment for methylation only and one assessing enrichment for expression only. A Reactome pathway was identified as over-represented in a community if its FDR was <0.05 in at least one of these three ORAs (Benjamini-Hochberg FDR as implemented in fgsea). Ten of the 59 communities of size ≥5 genes were enriched for at least one REACTOME pathway at FDR <0.05; complete results of the enrichment analysis are available in Supplementary Data 1. Here, we highlight two communities that illustrate DRAGON’s ability to provide unexpected insight into disease processes.

**Figure 7.**
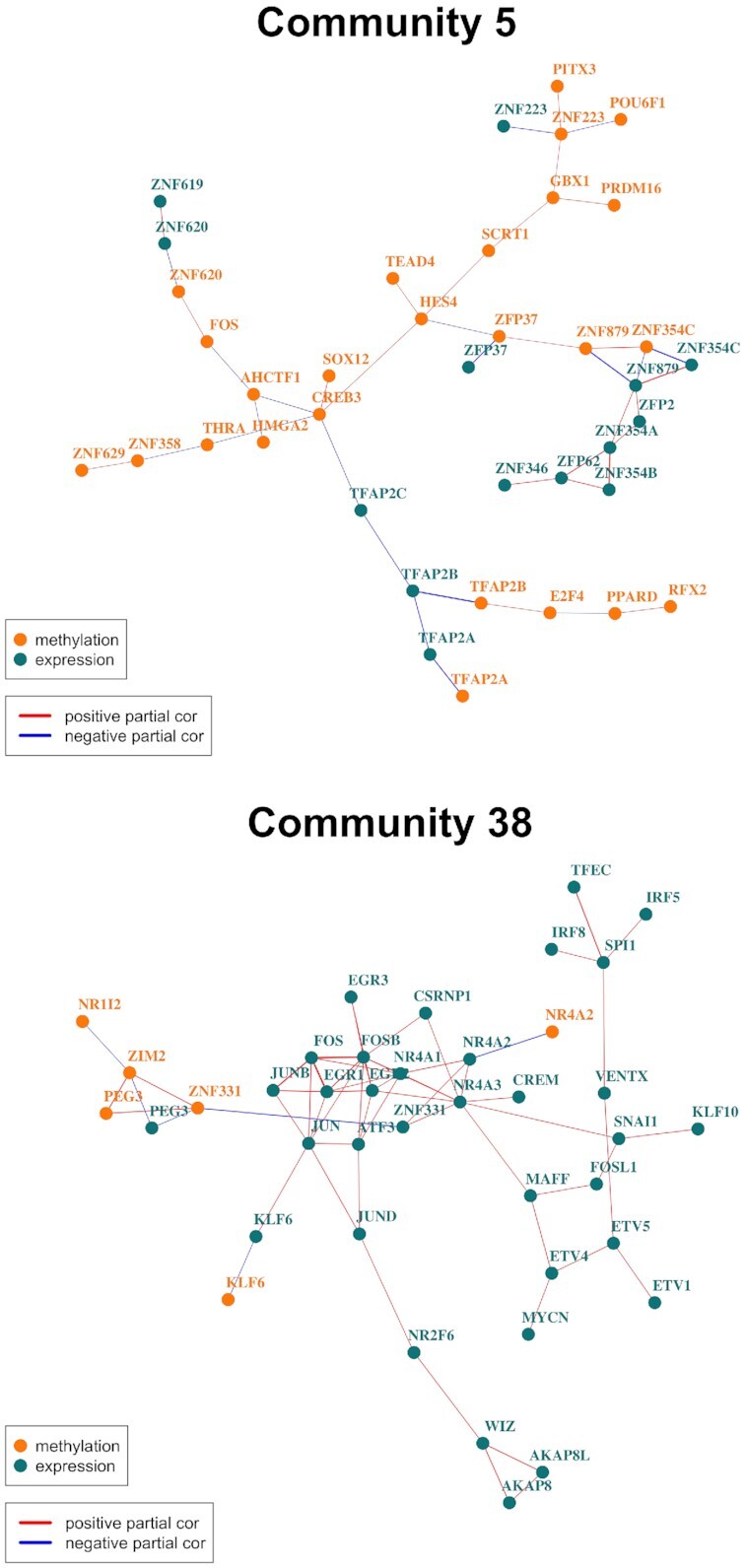
Example communities of interest in the DRAGON breast cancer gene expression-methylation network. Community 5 contains the TFAP2 family of transcription factors. Community 38 contains several hallmark cancer genes that are highly connected.

Community 5 consists of 39 TF nodes, 14 based on TF gene expression and 25 based on TF promoter methylation (Figure 7). The TF set in community 5 is enriched for several Reactome pathways related to the TFAP2 family of transcription factors (Supplementary Data 1). TFAP2C has been implicated in estrogen response signaling in breast cancer, which plays a major role in breast cancer development, progression, and therapeutic response (56); estrogen response signaling is also a key determinant of breast cancer molecular subtype (57).

To explore the role of the TFAP2 family in our DRAGON network, we obtained mRNA-based subtype classifications (basal, HER2, luminal A, luminal B and normal) for the tumor samples using the PanCancerAtlas_subtypes function from the TCGAbiolinks R package (58). We then evaluated subtype-specific methylation and expression among the TFAP2 genes represented in Community 5 (TFAP2A methylation and expression, TFAP2B methylation and expression, TFAP2C expression; Supplementary Figure S18).

TFAP2A and TFAP2B methylation both differed significantly based on subtype (Kruskal–Wallis test, TFAP2A *p* = 3.3 × 10^−4^; TFAP2B *p* < 2.2 × 10^−^^16^) as did TFAP2A, TFAP2B and TFAP2C expression (TFAP2A *p* = 1.9 × 10^−12^, TFAP2B *p* < 2.2 × 10^−^^16^, TFAP2C *p* < 1.9 × 10^−10^). However, TFAP2C methylation, which was notably excluded from this community, did not differ significantly between subtypes (*p* = 0.17). To illustrate the multi-omic phenotyping possible with DRAGON communities, we investigated TFAP2B further. The median TFAP2B promoter methylation beta level was 0.55 in basal samples versus 0.17 in non-basal samples; the median TFAP2B expression among basal samples was 0.49 TPM versus a median of 36.46 TPM in non-basal samples. This multi-omic phenotype of increased methylation and decreased expression in basal samples follows the canonical paradigm that promoter methylation results in gene silencing. To explore the predictive power of this joint information, we constructed a logistic regression model for multi-omic subtype classification, regressing basal vs. non-basal subtype against TFAP2B methylation, expression, and an interaction term between the two. In the resulting model, TFAP2B methylation and the interaction term between TFAP2B methylation and expression were both significant (methylation: OR = 147.76, 95 % CI: [44.21, 493.82]; methylation*expression: OR = 0.873, 95 % CI: [0.797, 0.957]) while TFAP2B expression was not (OR = 1.004, 95 % CI: [0.989, 1.018]). The AUROC of this model was 0.85; in contrast, the AUROC of a similar model using TFAP2B methylation alone as a predictor was 0.82 and using TFAP2B expression alone, 0.79, demonstrating the synergistic power of multi-omic features for class discrimination. Although this classification model does not outperform the expression-based subtype classification used by the TCGA, these results are nonetheless impressive given that they are based on two measurements of the omic state of a single gene.

Community 38 consists of 40 nodes, six of which represent promoter methylation and 34 of which represent gene expression (Figure 7). This community is enriched for 25 Reactome pathways, including signaling by receptor tyrosine kinases (FDR = 6.4 × 10^−9^), estrogen-dependent gene expression (FDR = 1.5 × 10^−3^), PTEN regulation (FDR = 0.02), and MAPK family signaling cascades (FDR = 0.02). The nodes primarily driving these enrichments include EGR1, EGR2, EGR3, FOS, FOSB, JUNB and JUND. These seven nodes comprise a tightly co-expressed subgraph of Community 38 and are well-known players in cancer signaling (59,60).

NR4A2 methylation and KLF6 methylation are both degree-one nodes in Community 38; their only edges indicate negative partial correlation with their own expression. It may be that differential methylation of NR4A2 and KLF6 drives subtype-specific activity in the pathways of this community by modulating the expression of NR4A2 and KLF6, which are connected via FOS and JUN expression to the eight-node cluster of coexpressed TFs. In comparing these nodes between subtypes, we found that both KLF6 and NR4A2 are significantly differentially methylated (KLF6: Kruskal–Wallis chi-squared = 93.04, df = 4, *P* < 2.2 × 10^−16^; NR4A2: Kruskal–Wallis chi-squared = 17.98, df = 4, *p* = 0.001; Supplementary Figure S19). To provide a benchmark for differential methylation between subtypes, we performed the same statistical test for a ‘housekeeping’ transcription factor (ATF1), which showed no significant difference in methylation (Kruskal–Wallis chi-squared = 6.62, df = 4, *p* = 0.158; Supplementary Figure S19), indicating that the differential methylation of these key transcription factors may play a substantial role in breast cancer beyond those already discussed, particularly given their association with the EGR/FOS/JUN expression cluster and the importance of these transcription factors in a wide range of cancer processes.

## CONCLUSION AND SUMMARY

Regulation of transcriptional processes in the cell involve multiple interacting partners that include transcription factors and their expression, regulation of their targets, and epigenetic factors such as DNA methylation that may enhance or disrupt regulatory interactions. Simple measures such as correlation fail to capture meaningful regulatory associations and can be dominated by spurious correlations between genes that are expressed at relatively low levels or that exhibit similar patterns of expression due to factors unrelated to the biological state of the system. Partial correlation analysis allows better discrimination between potentially causal associations between regulators and their regulatory targets and may lead to greater insight into the underlying biology of the systems we choose to study. The problem in conducting such an analysis is that different types of omics data often present with different scales, biases, and error distributions.

DRAGON is a partial correlation framework optimized for the integration of multiple ‘layers’ of omics data into a unified association network that allows us to understand both associations between biological variables such as gene expression and the potential drivers of the observed correlations. DRAGON is based on Gaussian Graphical Models (GGMs) and uses a regularization scheme to optimize the trade-off between the network’s complexity and its estimation accuracy while explicitly taking into account the distinct data characteristics of the various omics data types used in the model. DRAGON accounts for differences in edge densities and feature sizes, enabling improved estimation of partial correlations compared to layer-agnostic GGMs. The advantages of DRAGON are particularly evident in simulations when the number of variables *p* is the same order of magnitude or exceeds the sample size *n*, as is the case in nearly all omics experiments.

We recognize that DRAGON has some limitations. DRAGON’s GGM framework assumes multivariate normally distributed data which, for biological data, generally does not hold. For continuous distributions, data transformations such as the nonparanormal transformation (33) can be used to adjust input data to be approximately normally distributed; this approach, for example, allowed us to use DRAGON with methylation and gene expression data. However, other omics data types such as single-nucleotide polymorphism (SNP) data are categorical or ordinal, and alternative methods are needed to build these important regulatory elements into the DRAGON framework. Extending DRAGON to Mixed Graphical Models (61,62), which incorporate both continuous and discrete variables, could allow us to overcome this limitation of the current implementation.

It is also worth noting that DRAGON does not incorporate pre-defined network structures. Structured probabilistic graphical models have been studied fairly extensively (63–66) as they allow users to bias networks towards a given structure consistent with biological prior knowledge. Work by our group and others has demonstrated the power of introducing soft, knowledge-based constraints into optimization problems such as gene regulatory network inference (5,67). In the case of DRAGON, this could be achieved by *a priori* removing edges from the model based on known regulatory relationships by, for example, estimating the inverse biased covariance matrix with *a priori* specified zero entries (68). Alternatively, one could construct a model in which ‘established’ associations between elements are more likely to be included into the network by shifting their weights at initiation (64) or through the use of modified target matrices *T*^(*k*)^ in Equation (3). Both approaches, alone or in combination, would need to be carefully tested taking into account the effect that a bias on the network structure has on estimates of significance levels.

Although DRAGON represents an important step forward given our ability to collect increasingly large, multi-omic data sets, it is important to recognize that many problems in network inference are not addressed by DRAGON. Partial correlation relies on the data we have available, and many regulatory data types simply cannot be collected simultaneously and so remain hidden. Such hidden variables can lead to spurious associations and hamper the interpretation of networks in general. New technologies may provide additional omics layers, but integration of these will require additional methodological advances and the development of robust and scalable computational models.

Second, conditional independence is only one way to model associations in biological data. Conditional independence relationships encode the factorization properties of probability distributions and while appealing as a model, it is difficult to state definitively how this concept maps to the complexity of regulatory processes in biological systems. However, the same limitation holds for all measures of ‘relatedness’, comprising mutual exclusivity (9) and multivariate information measures (69). Elucidation of which type of similarity measure is most appropriate for inference of networks from biological data remains an open challenge (70), but probabilistic graphical models have been shown to perform well relative to other approaches (see the lasso regression methods in (70) as an example). To give a clear answer, experimental data together with the corresponding ground truth is optimally needed, but our use of inference methods underlies our present inability to determine the true regulatory processes that drive biological states. Consequently, method comparison of probabilistic graphical models that allows us to specify appropriate benchmarks with respect to edge recovery, maximum likelihood, and false discovery rate are the best available methods for testing the basic process of network inference and modeling, and assessment of biological insight gained trough model analysis remains a key element of validating new methods. On both accounts, DRAGON performs well giving limited data sets that reflect those typically available in omics studies of human health and disease. We also note that our analysis focused on observational data only, and the inferred networks are undirected. Statements on causality from observational data are difficult given correlation-based models, although it is possible to provide lower bounds on causal effects (71). However, our biological understanding of cause and effect can guide us, at least in part. Nevertheless, additional work needs to be done to address causality in the context of multi-omics and the use of structured approaches that incorporate prior causal knowledge may be an important next step. A simple adherence to the ‘central dogma of molecular biology,’ that DNA makes RNA, and RNA makes protein, could assist in defining causal relationships. Importantly, specifying a prior belief should not prohibit the inference of more complex regulatory mechanisms contradicting these beliefs: otherwise, we stand to discover only what we already know.

DRAGON represents a significant contribution to network inference by presenting a framework for modeling of partial correlations across multiple layers of omics data. DRAGON-estimated networks provide new insights into regulatory processes that may be overlooked by other methods as they are capable of identifying direct multi-omic relationships via a Gaussian graphical model framework. DRAGON is easy to use and freely available as open source software in the Network Zoo package (netZooPy v0.8; netzoo.github.io).

## DATA AVAILABILITY

DRAGON is available through the Network Zoo package (netZooPy v0.8; netzoo.github.io) and a vignette to demonstrate its use can be found in netBooks (netbooks.networkmedicine.org). Both are also available as supplementary material. Code to reproduce the TCGA breast cancer methylation analysis is available on Github at https://github.com/katehoffshutta/DRAGON-TCGA-BRCA (doi:10.5281/zenodo.7336963).

## Supplementary Material

gkac1157_Supplemental_Files

## Notes

Present address: Deborah Weighill, Lineberger Comprehensive Cancer Center, University of North Carolina at Chapel Hill, Chapel Hill, NC, USA.
