# Supplementary material for: DRAGON: Determining Regulatory Associations using Graphical models on multi-Omic Networks
Source: Nucleic Acids Res. 2022 Dec 19;51(3):e15. doi: 10.1093/nar/gkac1157 (PMC9943674; doi:10.1093/nar/gkac1157)

DRAGON example

Contents

- Load DRAGON
- Simulate data with two omics layers
- Regularization
- Estimate partial correlation matrix
- Assign significance levels

Load DRAGON

We first load functions from `dragon.py`:

```
from dragon import simulate_dragon_data, Scale, estimate_penalty_parameters_dragon
from dragon import get_partial_correlation_dragon, estimate_p_values_dragon
import matplotlib.pyplot as plt
import numpy as np
from mpl_toolkits.mplot3d import Axes3D, art3d
```

Simulate data with two omics layers

To start, we use `simulate_dragon_data` to simulate multi-omics data. Here, `p1` and `p2`refers to the number of variables in omics layer 1 and 2, respectively. The proportion of edges within layer 1 and 2 is set by `eta11` and `eta22`, respectively, and the proportion of edges which connect variables from layer 1 with variables from layer 2 is `eta12`. The sample size is given by `n`, the noise by `epsilon`, and the seed by `seed`.

```
n = 1000
p1 = 500
p2 = 100

X1, X2, Theta, _ = simulate_dragon_data(eta11=0.005, eta12=0.005, eta22=0.05,
                                        p1=p1, p2=p2, epsilon=[0.1,0.1],
                                        n=n, seed=123)
```

This returns data from omics layer 1, `X1`, and from layer 2, `X2`, with `n` measurements in the rows and with `p1` and `p2` variables in the columns. `Theta` corresponds to the underlying, true precision matrix. First, we standardize the variables (DRAGON was verified for standardized data) and verify the dimensions of `X1` and `X2`:

```
X1 = Scale(X1)
X2 = Scale(X2)
print(X1.shape)
print(X2.shape)
```

(1000, 500)
(1000, 100)

Regularization

DRAGON uses an efficient way to estimate regularization parameters:

```
lambdas, lambdas_landscape = estimate_penalty_parameters_dragon(X1, X2)
print(lambdas)
```

(0.8230129199774914, 0.7163033748406636)

We can also visualize the regularization landscape as follows.

```
fig = plt.figure()
ax = plt.axes(projection='3d')
x = np.arange(0,1.01,0.01)
ax.contour3D(x, x, lambdas_landscape, 50, cmap='binary')
ind = np.unravel_index(np.argmin(lambdas_landscape, axis=None),
                       lambdas_landscape.shape)
ax.plot([x[int(ind[1])]], [x[int(ind[0])]],
        [lambdas_landscape[int(ind[0]),int(ind[1])]],
        markerfacecolor='k', markeredgecolor='r', marker='o',
        markersize=7, alpha=1)
ax.set_xlabel('$\lambda_1$', fontsize=16)
ax.set_ylabel('$\lambda_2$', labelpad=15, fontsize=16)
plt.show()
```

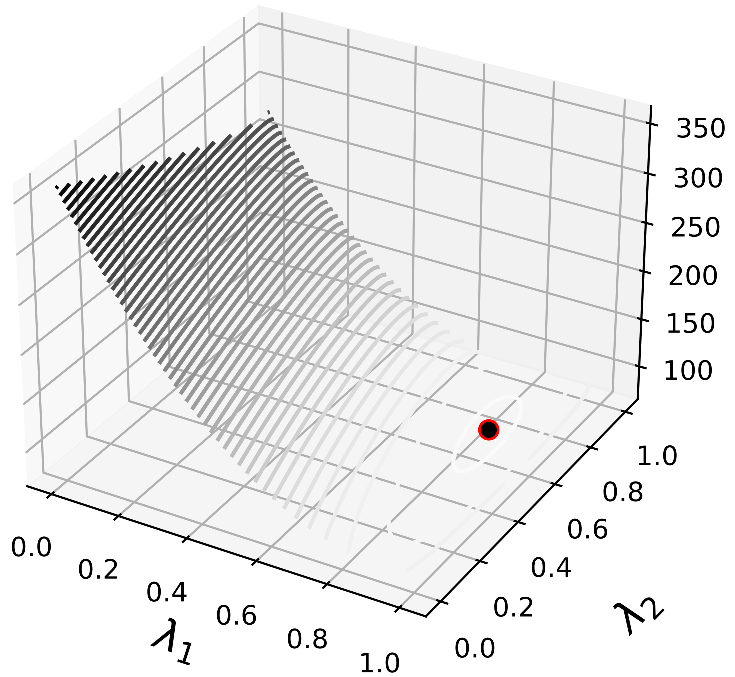

Estimate partial correlation matrix

Next, we estimate DRAGON's shrunken partial correlations using the regularization parameters determined above.

```
r = get_partial_correlation_dragon(X1, X2, lambdas)
```

Assign significance levels

Finally, we assign significance levels. Here, we need to correctly specify `n`, `p1` and `p2`, and we have to use exactly the values `lambdas` that we used to estimate `r`. The first matrix contains p-values adjusted for multiple testing (BH) and the second the raw p-values.

```
adj_p_vals, p_vals = estimate_p_values_dragon(r, n, p1, p2, lambdas)
```

We can visualize the original precision matrix by

```
plt.imshow(Theta[0:20,0:20])
plt.colorbar()
plt.show()
```

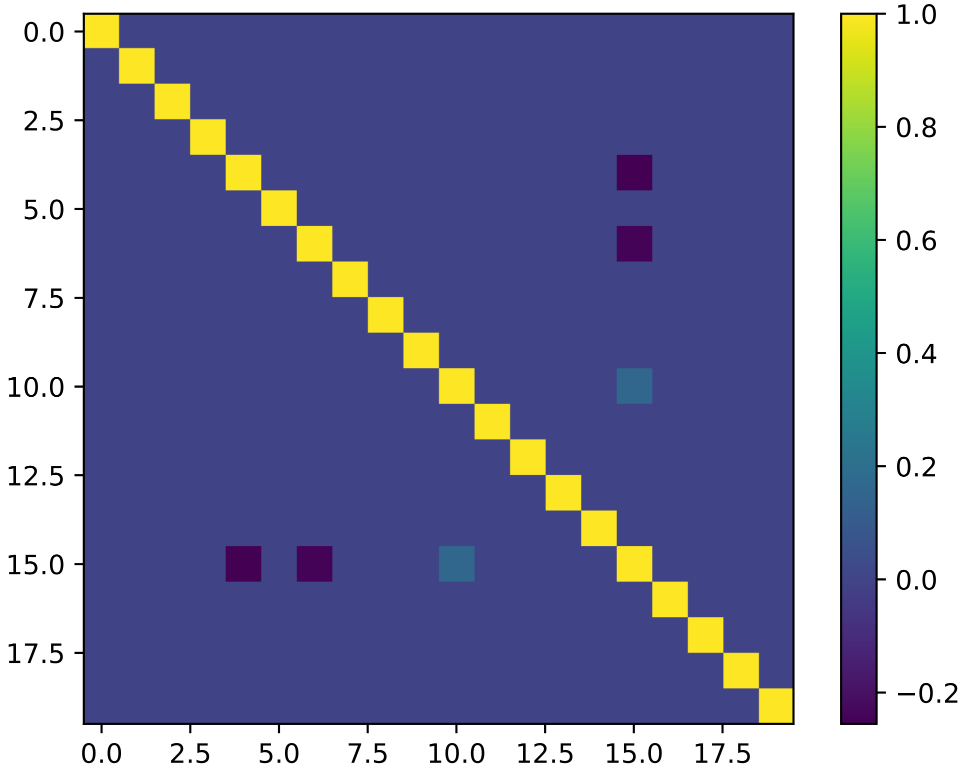

and the adjusted significance levels of estimated edges by

```
plt.imshow(adj_p_vals[0:20,0:20])
plt.colorbar()
plt.show()
```

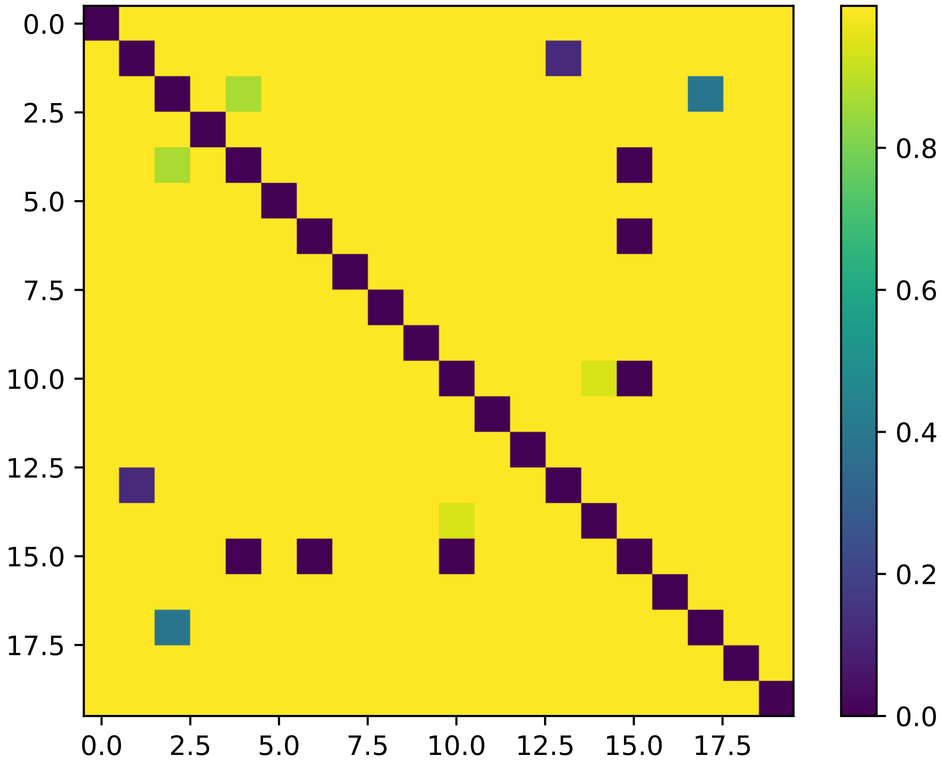

Supplement: gkac1157_Supplemental_Files [file gkac1157_supplemental_files.zip › DRAGON_example.pdf]
