# Supplementary material for: DRAGON: Determining Regulatory Associations using Graphical models on multi-Omic Networks
Source: Nucleic Acids Res. 2022 Dec 19;51(3):e15. doi: 10.1093/nar/gkac1157 (PMC9943674; doi:10.1093/nar/gkac1157)
Supplement: gkac1157_Supplemental_Files [file gkac1157_supplemental_files.zip › dragon_supplement.pdf]

Supplementary Material

DRAGON: Determining Regulatory Associations using Graphical models on multi-Omic Networks

1. Analytical estimates for regularization parameters λ_1 and λ_2

Equation 8 of the main manuscript decomposes the quantity

$$R = E\left[||\hat{\Sigma} - \Sigma||_F^2\right] \tag{1}$$

into terms which can be efficiently calculated analytically, yielding estimates for optimal regularization parameters λ_1 and λ_2 of DRAGON in the 2-omic case. Here, we derive this equation.

Notation

Let $\Sigma = \{\sigma_{ij}\}$ be the true population covariance and let $S = \{s_{ij}\}$ be sample covariance, i.e., the usual maximum likelihood estimator for Σ. Next, let $\hat{\Sigma} = \{\hat{\sigma}_{ij}\}$ be the covariance shrinkage estimator defined in the main manuscript, i.e.:

$$\hat{\Sigma} = \{\hat{\sigma}_{ij}\} = \begin{pmatrix} (1-\lambda_1)S^{(1,1)} & \sqrt{1-\lambda_1}\sqrt{1-\lambda_2}S^{(1,2)} \\ \sqrt{1-\lambda_2}\sqrt{1-\lambda_1}S^{(2,1)} & (1-\lambda_2)S^{(2,2)} \end{pmatrix} + \begin{pmatrix} \lambda_1 \mathrm{diag}(S^{(1,1)}) & 0 \\ 0 & \lambda_2 \mathrm{diag}(S^{(2,2)}) \end{pmatrix} \tag{2}$$

where we have divided S into blocks consisting of $S^{(1,1)}$, the sample covariance of omics layer 1; $S^{(2,2)}$, the sample covariance of omics layer 2; and $S^{(1,2)} = t(S^{(2,1)})$, the sample covariance between omics layer 1 and omics layer 2. Finally, let $(i,j)^{(A,B)}$ represent the row and column indices in the (A,B) block of the matrices Σ and S.

Derivation

We derive an expression for R in terms of λ_1 and λ_2 by considering three cases:

(i) i, j such that σ_{ij} represents the within-omic covariance of two variables i and j, both in omics layer 1. The notation we use for these indices is $(i,j)^{(1,1)}$.

(ii) i, j such that σ_{ij} represents the within-omic covariance of two variables i and j, both in omics layer 2. The notation we use for these indices is $(i,j)^{(2,2)}$.

(iii) i, j such that σ_{ij} represents the between-omic covariance of variable i, which is in omics layer 1, and variable j, which is in omics layer 2.The notation we use for these indices is $(i,j)^{(1,2)}$.

In deriving these steps, we partition R into the sum of four components: $R^{(1,1)}$, obtained from $\Sigma^{(1,1)}$ and $S^{(1,1)}$; $R^{(2,2)}$, obtained from $\Sigma^{(2,2)}$ and $S^{(2,2)}$; $R^{(1,2)}$, obtained from $\Sigma^{(1,2)}$ and $S^{(1,2)}$; and $R^{(2,1)}$, obtained from $\Sigma^{(2,1)}$ and $S^{(2,1)}$. From these, we can write the final equation

$$\begin{aligned} R &= R^{(1,1)} + R^{(2,2)} + R^{(1,2)} + R^{(2,1)} \\ &= R^{(1,1)} + R^{(2,2)} + 2R^{(1,2)} \end{aligned}$$

where the last line arises due to the symmetric nature of the covariance matrix and the Frobenius norm.

Case (i): both variables in omics layer 1

In this case, we have

$$\begin{aligned}
R^{(1,1)} = E\left[||\hat{\Sigma}^{(1,1)} - \Sigma^{(1,1)}||_F^2\right] &= \sum_{(i,j)^{(1,1)}} E\left[(\hat{\sigma}_{ij} - \sigma_{ij})^2\right] \\
&= \sum_{(i=j)^{(1,1)}} E\left[\left((1-\lambda_1)s_{ij} + \lambda_1 s_{ij} - \sigma_{ij}\right)^2\right] + \sum_{(i\neq j)^{(1,1)}} E\left[\left((1-\lambda_1)s_{ij} - \sigma_{ij}\right)^2\right] \\
&= \sum_{(i=j)^{(1,1)}} E\left[\left(s_{ij} - \sigma_{ij}\right)^2\right] + \sum_{(i\neq j)^{(1,1)}} E\left[\left((1-\lambda_1)s_{ij} - \sigma_{ij}\right)^2\right]
\end{aligned}$$

Next, we use the standard identity:

$$\begin{aligned}
Var[U] &= E[U^2] - (E[U])^2 \\
E[U^2] &= Var[U] + (E[U])^2
\end{aligned}$$

First, when $i \neq j$, let $U = s_{ij} - \sigma_{ij}$. Then we have

$$\begin{aligned}
R^{(1,1)} &= \sum_{(i=j)^{(1,1)}} Var[s_{ij} - \sigma_{ij}] + (E[s_{ij} - \sigma_{ij}])^2 + \sum_{(i\neq j)^{(1,1)}} E\left[\left((1-\lambda_1)s_{ij} - \sigma_{ij}\right)^2\right] \\
&= \sum_{(i=j)^{(1,1)}} Var[s_{ij}] + \sum_{(i\neq j)^{(1,1)}} E\left[\left((1-\lambda_1)s_{ij} - \sigma_{ij}\right)^2\right]
\end{aligned}$$

where the latter line arises because s_{ij} is an unbiased estimator of σ_{ij}. Next, we consider the term when $i \neq j$, letting $U = (1-\lambda_1)s_{ij} - \sigma_{ij}$.

$$\begin{aligned}
R^{(1,1)} &= \sum_{(i=j)^{(1,1)}} Var[s_{ij}] + \sum_{(i\neq j)^{(1,1)}} Var[(1-\lambda_1)s_{ij} - \sigma_{ij}] + (E[(1-\lambda_1)s_{ij} - \sigma_{ij}])^2 \\
&= \sum_{(i=j)^{(1,1)}} Var[s_{ij}] + \sum_{(i\neq j)^{(1,1)}} (1-\lambda_1)^2 Var[s_{ij}] + ((1-\lambda_1)E[s_{ij}] - \sigma_{ij})^2 \\
&= \sum_{(i=j)^{(1,1)}} Var[s_{ij}] + \sum_{(i\neq j)^{(1,1)}} (1-\lambda_1)^2 Var[s_{ij}] + \lambda_1^2 (E[s_{ij}])^2 \\
&= \sum_{(i=j)^{(1,1)}} Var[s_{ij}] + \sum_{(i\neq j)^{(1,1)}} Var[s_{ij}] - 2\lambda_1 Var[s_{ij}] + \lambda_1^2 Var[s_{ij}] + \lambda_1^2 (E[s_{ij}])^2 \\
&= \sum_{(i,j)^{(1,1)}} Var[s_{ij}] + \sum_{(i\neq j)^{(1,1)}} -2\lambda_1 Var[s_{ij}] + \lambda_1^2 E[s_{ij}^2]
\end{aligned}$$

Case (ii): both omics variables in layer 2

In this case, we can follow the analogy to the argument above to arrive at

$$R^{(2,2)} = \sum_{(i,j)^{(2,2)}} Var[s_{ij}] + \sum_{(i \neq j)^{(2,2)}} -2\lambda_2 Var[s_{ij}] + \lambda_2^2 E[s_{ij}^2] \tag{3}$$

Case (iii): variable *i* is in omics layer 1 and variable *j* is in omics layer 2.

In this case, we derive $R^{(1,2)}$, noting that $R^{(2,1)} = R^{(1,2)}$ by symmetry.

$$\begin{aligned}
R^{(1,2)} &= E\left[||\hat{\Sigma}^{(1,2)} - \Sigma^{(1,2)}||_F^2\right] \\
&= \sum_{(i,j)^{(1,2)}} E\left[(\hat{\sigma}_{ij} - \sigma_{ij})^2\right] \\
&= \sum_{(i,j)^{(1,2)}} Var[\hat{\sigma}_{ij} - \sigma_{ij}] + (E[\hat{\sigma}_{ij} - \sigma_{ij}])^2 \\
&= \sum_{(i,j)^{(1,2)}} (1-\lambda_1)(1-\lambda_2)Var[s_{ij}] + (E[\hat{\sigma}_{ij} - s_{ij} + s_{ij} - \sigma_{ij}])^2 \\
&= \sum_{(i,j)^{(1,2)}} (1-\lambda_1)(1-\lambda_2)Var[s_{ij}] + (E[\sqrt{1-\lambda_1}\sqrt{1-\lambda_2}s_{ij} - s_{ij}] + E[s_{ij} - \sigma_{ij}])^2 \\
&= \sum_{(i,j)^{(1,2)}} (1-\lambda_1)(1-\lambda_2)Var[s_{ij}] + (\sqrt{1-\lambda_1}\sqrt{1-\lambda_2} - 1)^2 (E[s_{ij}])^2
\end{aligned}$$

Now the expression is in terms of the variance and expectation of the sample covariance, which we can easily approximate with moment estimators. We continue manipulating the expression:

$$\begin{aligned}
R^{(1,2)} &= \sum_{(i,j)^{(1,2)}} \{1-\lambda_1-\lambda_2+\lambda_1\lambda_2\} Var[s_{ij}] + \left\{(1-\lambda_1)(1-\lambda_2) - 2\sqrt{1-\lambda_1}\sqrt{1-\lambda_2} + 1\right\} (E[s_{ij}])^2 \\
&= \sum_{(i,j)^{(1,2)}} \{1-\lambda_1-\lambda_2+\lambda_1\lambda_2\} Var[s_{ij}] + \left\{2-\lambda_1-\lambda_2+\lambda_1\lambda_2 - 2\sqrt{1-\lambda_1}\sqrt{1-\lambda_2}\right\} (E[s_{ij}])^2
\end{aligned}$$

Next, we again apply the identity $(E[U])^2 = E[U^2] - Var[U]$:

$$
\begin{aligned}
&= \sum_{(i,j)^{(1,2)}} \{1-\lambda_1-\lambda_2+\lambda_1\lambda_2\} Var[s_{ij}] + \left\{2-\lambda_1-\lambda_2+\lambda_1\lambda_2-2\sqrt{1-\lambda_1}\sqrt{1-\lambda_2}\right\}(E[s_{ij}^2]-Var[s_{ij}]) \\
&= \sum_{(i,j)^{(1,2)}} \left\{1-\lambda_1-\lambda_2+\lambda_1\lambda_2-(2-\lambda_1-\lambda_2+\lambda_1\lambda_2-2\sqrt{1-\lambda_1}\sqrt{1-\lambda_2})\right\} Var[s_{ij}] \\
&+ \left\{2-\lambda_1-\lambda_2+\lambda_1\lambda_2-2\sqrt{1-\lambda_1}\sqrt{1-\lambda_2}\right\} E[s_{ij}^2] \\
&= \sum_{(i,j)^{(1,2)}} \left\{-1+2\sqrt{1-\lambda_1}\sqrt{1-\lambda_2}\right\} Var[s_{ij}] + \left\{2-\lambda_1-\lambda_2+\lambda_1\lambda_2-2\sqrt{1-\lambda_1}\sqrt{1-\lambda_2}\right\} E[s_{ij}^2] \\
&= -\sum_{(i,j)^{(1,2)}} Var[s_{ij}] + 2\sqrt{1-\lambda_1}\sqrt{1-\lambda_2} \sum_{(i,j)^{(1,2)}} (Var[s_{ij}]-E[s_{ij}^2]) + (2-\lambda_1-\lambda_2+\lambda_1\lambda_2) \sum_{(i,j)^{(1,2)}} E[s_{ij}^2])
\end{aligned}
$$

Combining cases (i), (ii), and (iii)

Our final expression for R is

$$
\begin{aligned}
R &= R^{(1,1)} + R^{(2,2)} + 2R^{(1,2)} \\
&= \sum_{(i,j)^{(1,1)}} Var[s_{ij}] + \sum_{(i\neq j)^{(1,1)}} -2\lambda_1 Var[s_{ij}] + \lambda_1^2 E[s_{ij}^2] \\
&+ \sum_{(i,j)^{(2,2)}} Var[s_{ij}] + \sum_{(i\neq j)^{(2,2)}} -2\lambda_2 Var[s_{ij}] + \lambda_2^2 E[s_{ij}^2] \\
&+ 2*\left\{ -\sum_{(i,j)^{(1,2)}} Var[s_{ij}] + 2\sqrt{1-\lambda_1}\sqrt{1-\lambda_2} \sum_{(i,j)^{(1,2)}} (Var[s_{ij}]-E[s_{ij}^2]) + (2-\lambda_1-\lambda_2+\lambda_1\lambda_2) \sum_{(i,j)^{(1,2)}} E[s_{ij}^2]) \right\} \\
&= \sum_{(i\neq j)^{(1,1)}} -2\lambda_1 Var[s_{ij}] + \lambda_1^2 E[s_{ij}^2] + \sum_{(i,j)^{(2,2)}} Var[s_{ij}] - 2\lambda_2 Var[s_{ij}] + \lambda_2^2 E[s_{ij}^2] \\
&+ 4\sqrt{1-\lambda_1}\sqrt{1-\lambda_2} \sum_{(i,j)^{(1,2)}} (Var[s_{ij}]-E[s_{ij}^2]) \\
&+ 2*(2-\lambda_1-\lambda_2+\lambda_1\lambda_2) \sum_{(i,j)^{(1,2)}} E[s_{ij}^2]
\end{aligned}
$$

Combining like terms for functions of λ_1 and λ_2, we can write

$$
\begin{aligned}
R &= \sum_{(i\neq j)^{(1,1)}} -2\lambda_1 Var[s_{ij}] + \lambda_1^2 E[s_{ij}^2] + \sum_{(i\neq j)^{(2,2)}} -2\lambda_2 Var[s_{ij}] + \lambda_2^2 E[s_{ij}^2] \\
&+ 4\sqrt{1-\lambda_1}\sqrt{1-\lambda_2} \sum_{(i,j)^{(1,2)}} (Var[s_{ij}] - E[s_{ij}^2]) \\
&+ 2*(2-\lambda_1-\lambda_2+\lambda_1\lambda_2) \sum_{(i,j)^{(1,2)}} E[s_{ij}^2] \\
&= 4 \sum_{(i,j)^{(1,2)}} E[s_{ij}^2] \\
&+ \lambda_1 \left\{ -2 \sum_{(i\neq j)^{(1,1)}} Var[s_{ij}] - 2 \sum_{(i,j)^{(1,2)}} E[s_{ij}^2] \right\} \\
&+ \lambda_2 \left\{ -2 \sum_{(i\neq j)^{(2,2)}} Var[s_{ij}] - 2 \sum_{(i,j)^{(1,2)}} E[s_{ij}^2] \right\} \\
&+ \lambda_1^2 \sum_{(i\neq j)(1,1)} E[s_{ij}^2] \\
&+ \lambda_2^2 \sum_{(i\neq j)^{(2,2)}} E[s_{ij}^2] \\
&+ \lambda_1\lambda_2 \left\{ 2 \sum_{(i,j)^{(1,2)}} E[s_{ij}^2] \right\} \\
&+ \sqrt{1-\lambda_1}\sqrt{1-\lambda_2} \left\{ 4 \sum_{(i,j)^{(1,2)}} (Var[s_{ij}] - E[s_{ij}^2]) \right\}
\end{aligned}
$$

This expression is of the form

$$
R = \text{const.} + \lambda_1 T_1^{(1)} + \lambda_2 T_1^{(2)} + \lambda_1^2 T_2^{(1)} + \lambda_2^2 T_2^{(2)} + \lambda_1\lambda_2 T_3 + \sqrt{1-\lambda_1}\sqrt{1-\lambda_2} T_4 \tag{4}
$$

where the first term is a constant with respect to λ_1 and λ_2 and the remaining terms T are defined as:

$$
\begin{aligned}
T_1^{(k)} &= -2 \left(\sum_{(i\neq j)^{(k,k)}} Var[s_{ij}] + \sum_{(i,j)^{(1,2)}} E[s_{ij}^2] \right); k = 1,2 \\
T_2^{(k)} &= \sum_{(i\neq j)^{(k,k)}} E[s_{ij}^2]; k = 1,2 \\
T_3 &= 2 \sum_{(i,j)^{(1,2)}} E[s_{ij}^2] \\
T_4 &= 4 \sum_{(i,j)^{(1,2)}} (Var[s_{ij}] - E[s_{ij}^2])
\end{aligned}
$$

2. Simulation study to illustrate partial correlations

We first simulated normal distributed values of the "regulator" via $regulator \sim N(\mu = 0, \sigma = 1)$. Then we defined gene A and B to equal $regulator + \varepsilon_{A,B}$, respectively, where $\varepsilon_{A,B} \sim N(\mu = 0, \sigma = .1)$. Simulations were performed using the R programming environment version 4.1.2 [1]. For ordinary partial correlation estimates, we used the R package "ppcor" [2].

3. Supplementary figures for simulation studies

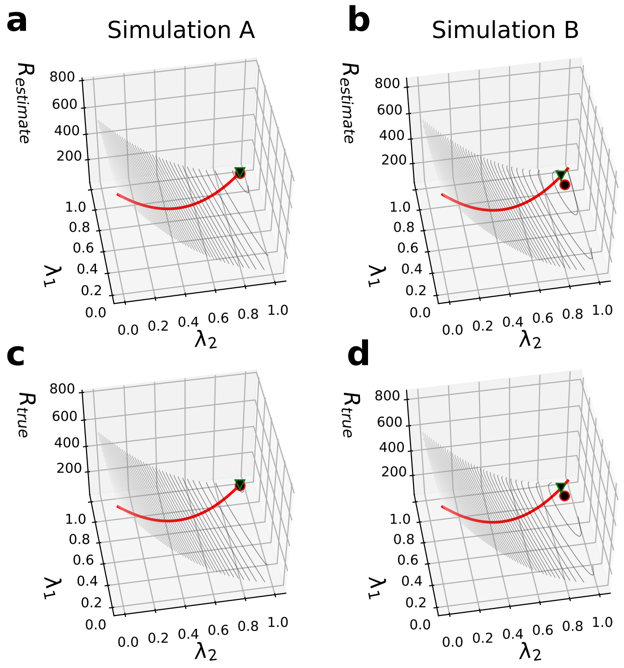

Figure S1: Parameter landscapes for DRAGON in studies A and B. The plot is understood analogous to Figure 2 in the main article but now for $n = 500$.

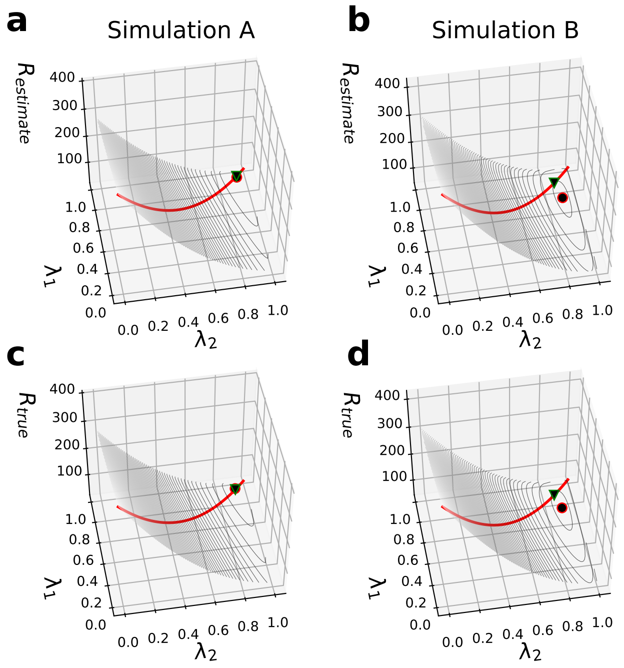

Figure S2: Parameter landscapes for DRAGON in studies *A* and *B*. The plot is understood analogous to Figure 2 in the main article but now for $n = 1,000$.

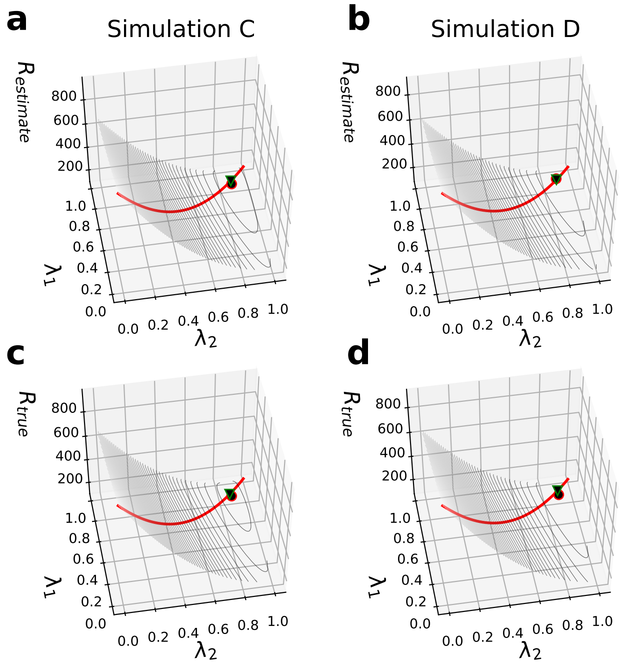

Figure S3: Parameter landscapes for DRAGON. Estimated and true R (upper and lower row) in dependency of λ_1 and λ_2 in simulation studies C (left column) and D (right column) for $n = 500$. Figures a and b show the estimated R for studies C and D, respectively. Figures c and d show the corresponding ground truth. The red circles indicate the minima for each plot in the λ_1–λ_2 plane, and the green triangles give the minima on the diagonal R values shown in red, corresponding to the standard GGM.

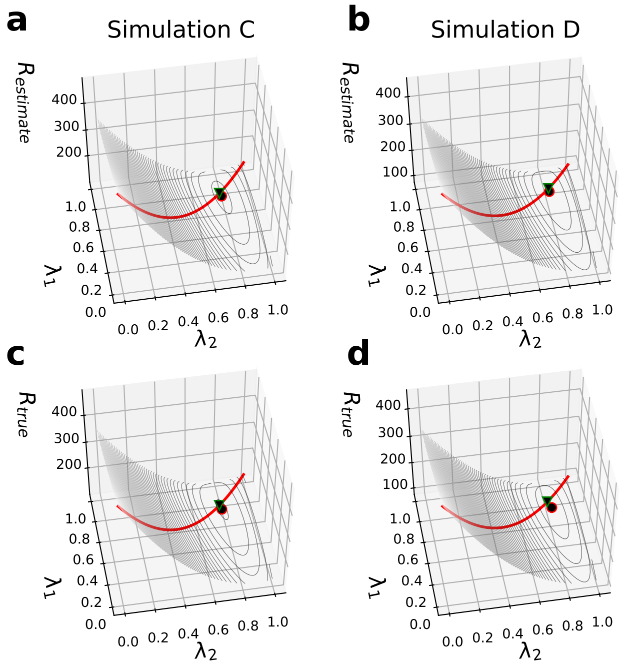

Figure S4: Parameter landscapes for DRAGON in studies C and D. The plot is understood analogous to Figure S5 but now for $n = 1,000$.

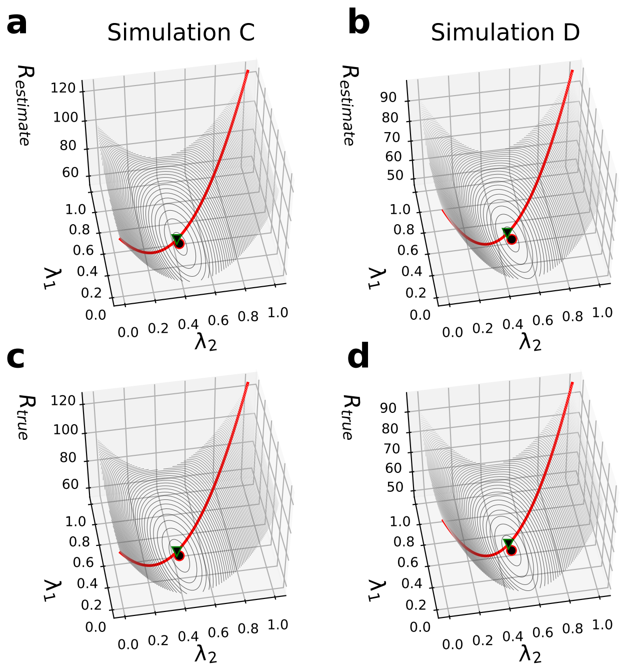

Figure S5: Parameter landscapes for DRAGON in studies C and D. The plot is understood analogous to Figure S5 but now for $n = 5,000$.

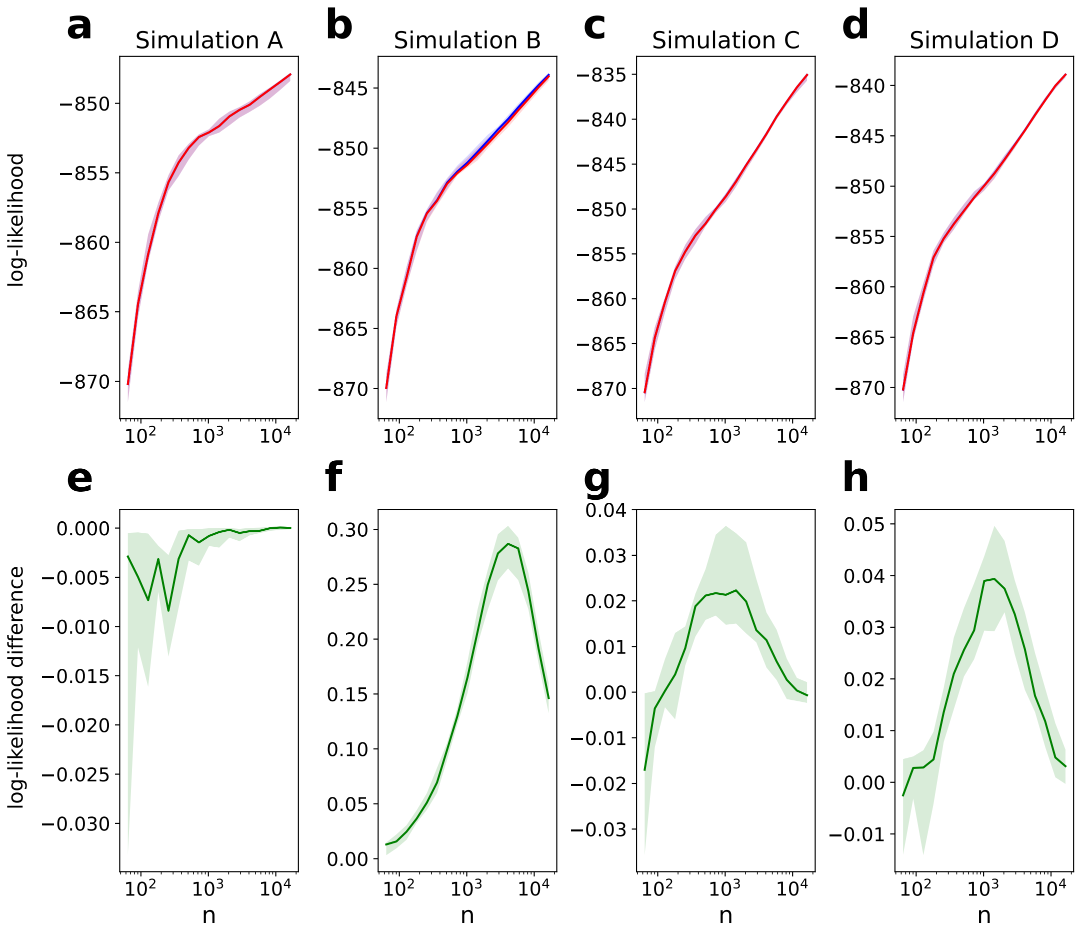

Figure S6: Log-likelihood comparison between DRAGON and GGM. The upper row, Figures a to d give the log-likelihood evaluated on test data versus the number of training samples, n, for simulation studies A to D, respectively. Results from DRAGON are given in blue and those from the GGM in red. The lines correspond to the median log-likelihood and the bands to the 25% and 75% percentiles of the distribution. Figures e to h show the corresponding log-likelihood differences (DRAGON minus GGM) in green.

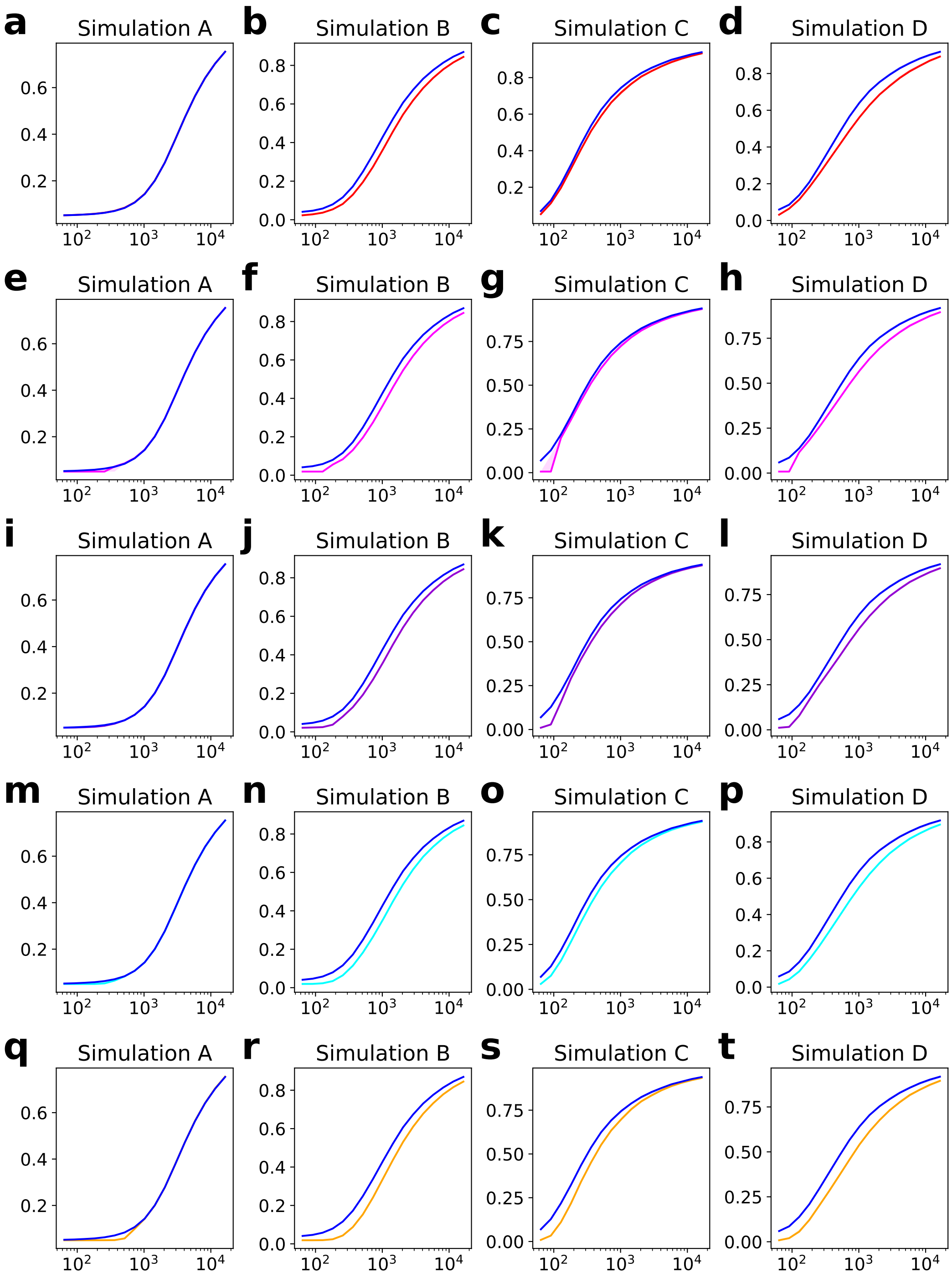

Figure S7: Area under the precision-recall curve (AUC-PR) for edge-recovery performance versus sample size n of simulation studies A to D (columns). Each row compares DRAGON (blue) with GGM (red, Fig. a to d), GeneNet (magenta, Fig. e to h), B-NW-SL (purple, Fig i to l), D-S-NW-SL (cyan, Fig. m to p), and D-S-GL (orange, Fig. q to t), respectively. The lines correspond to the median AUC-PR and the bands to the 25% and 75% percentiles of the distribution.

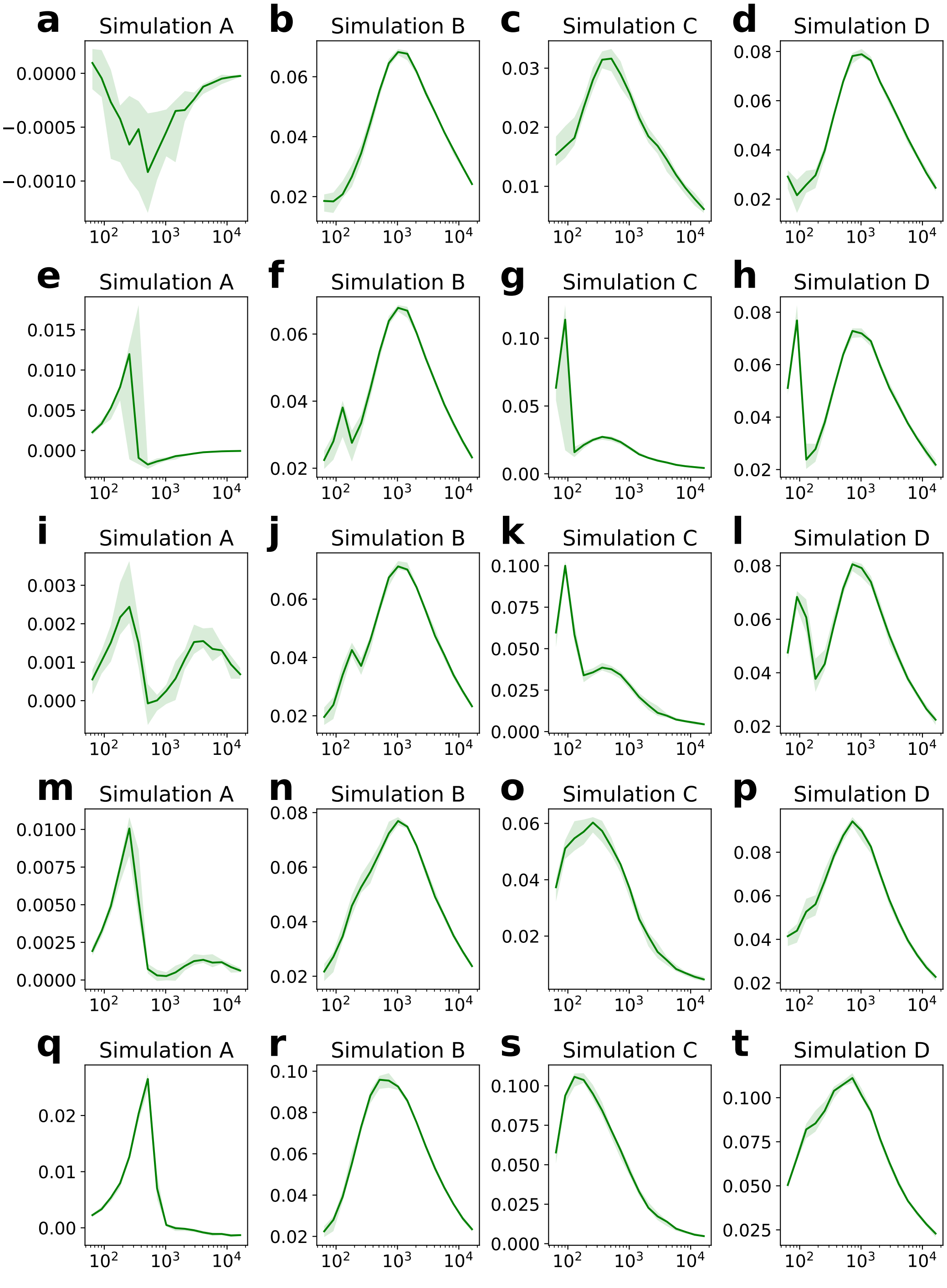

Figure S8: Area under the precision-recall curve (AUC-PR) differences versus sample size n of simulation studies A to D (columns). Each row corresponds to the AUC-PR difference DRAGON minus GGM (Fig. a to d), DRAGON minus GeneNet (Fig. e to h), DRAGON minus B-NW-SL (Fig. i to l), DRAGON minus D-S-NW-SL (Fig. m to p), and DRAGON minus D-S-GL (Fig. q to t), respectively. The green lines correspond to the median AUC-PR and the bands to the 25% and 75% percentiles of the distribution.

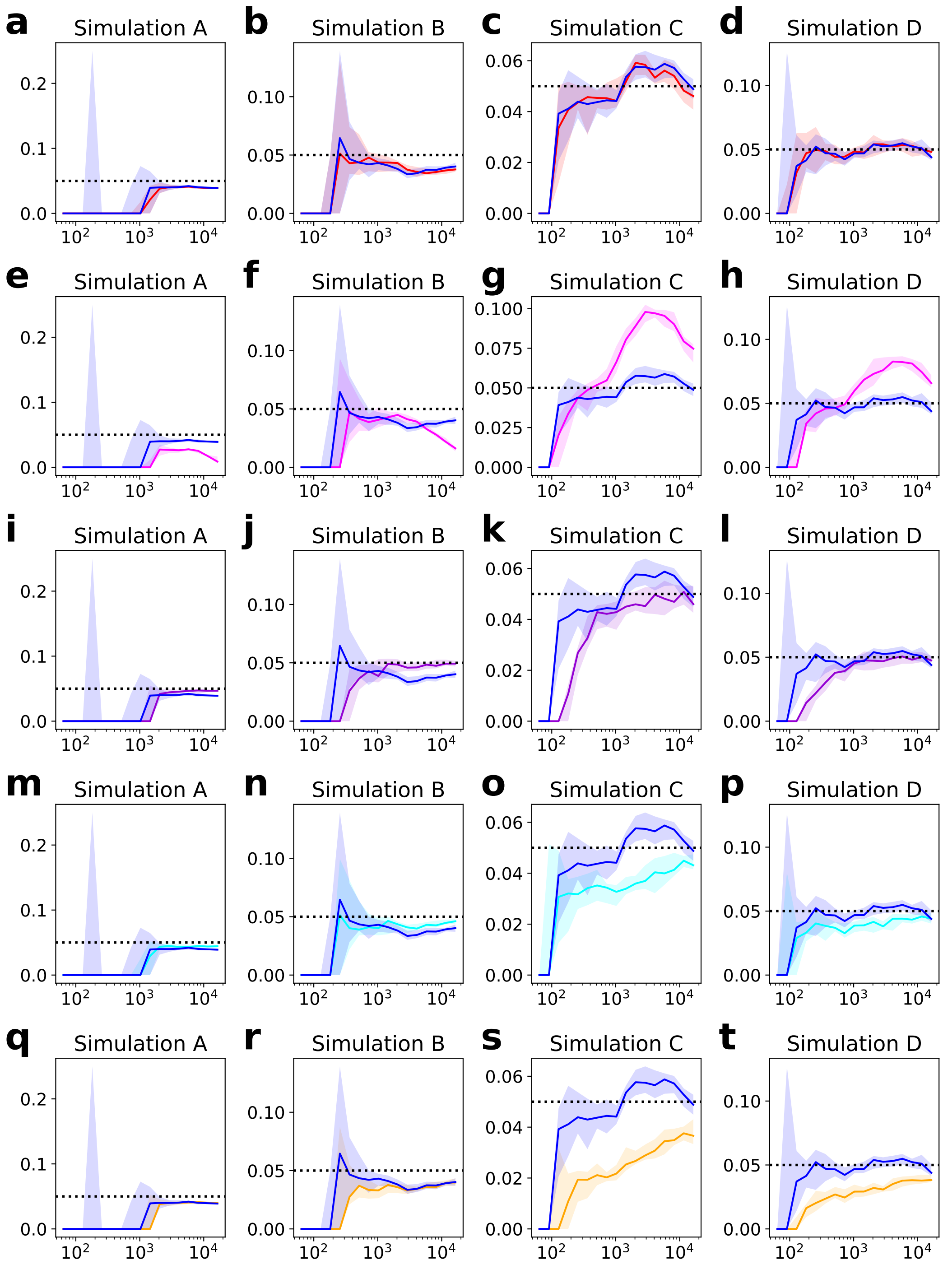

Figure S9: Posterior False-discovery rates (FDRs) versus sample size n of simulation studies A to D (columns) for significance threshold $\alpha = 0.05$ (dotted line). Each row compares DRAGON (blue) with GGM (red, Fig. a to d), GeneNet (magenta, Fig. e to h), B-NW-SL (purple, Fig. i to l), D-S-NW-SL (cyan, Fig. m to p), and D-S-GL (orange, Fig. q to t), respectively. The lines correspond to the median FDR and the bands to the 25% and 75% percentiles of the distribution. If no edges were called, the FDR was set to zero.

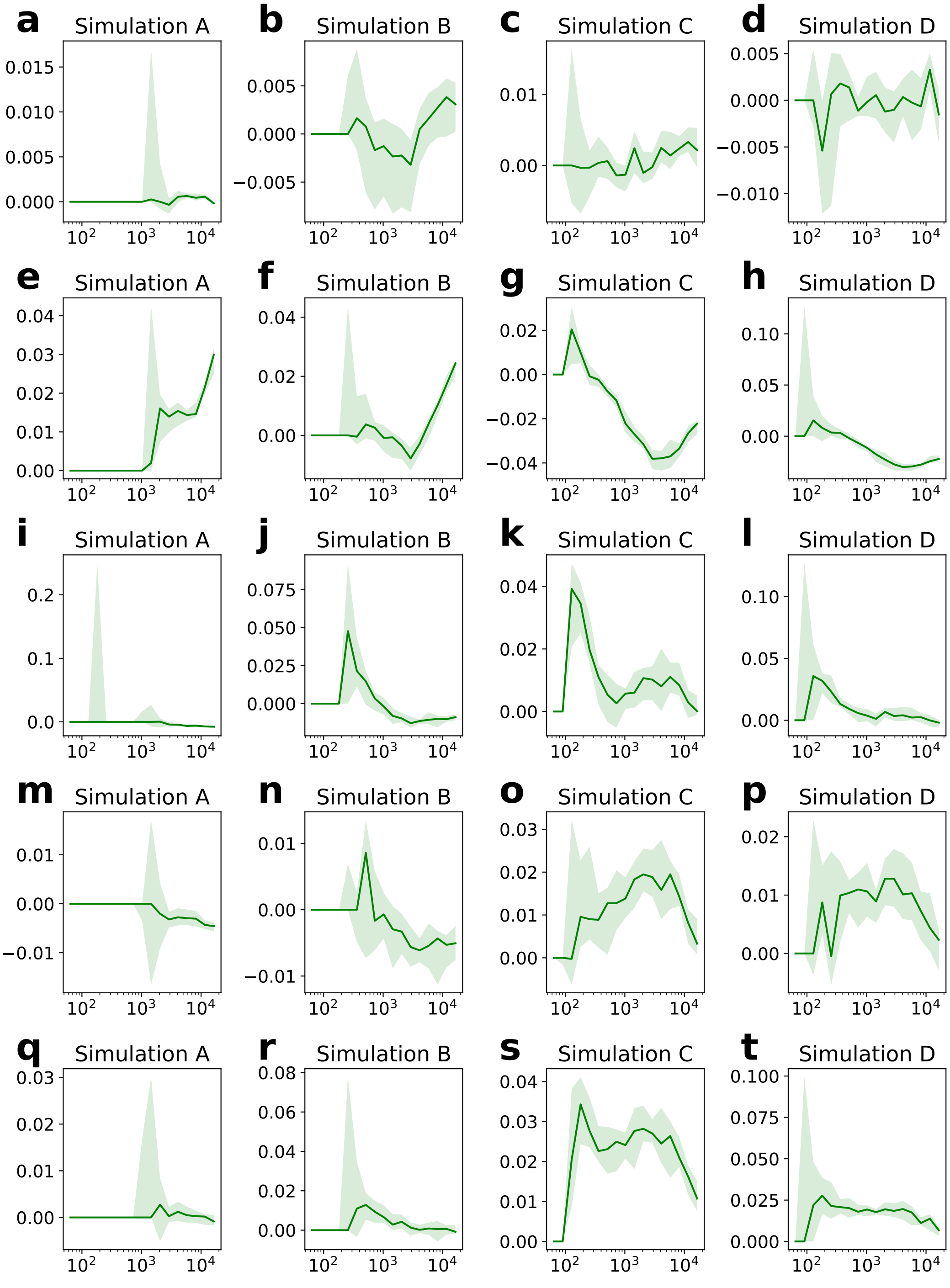

Figure S10: False-discovery rate (FDR) differences versus sample size n of simulation studies A to D (columns). Each row corresponds to the FDR difference DRAGON minus GGM (Fig. a to d), DRAGON minus GeneNet (Fig. e to h), DRAGON minus B-NW-SL (Fig. i to l), DRAGON minus D-S-NW-SL (Fig. m to p), and DRAGON minus D-S-GL (Fig. q to t), respectively. The green lines correspond to the median FDR and the bands to the 25% and 75% percentiles of the distribution.

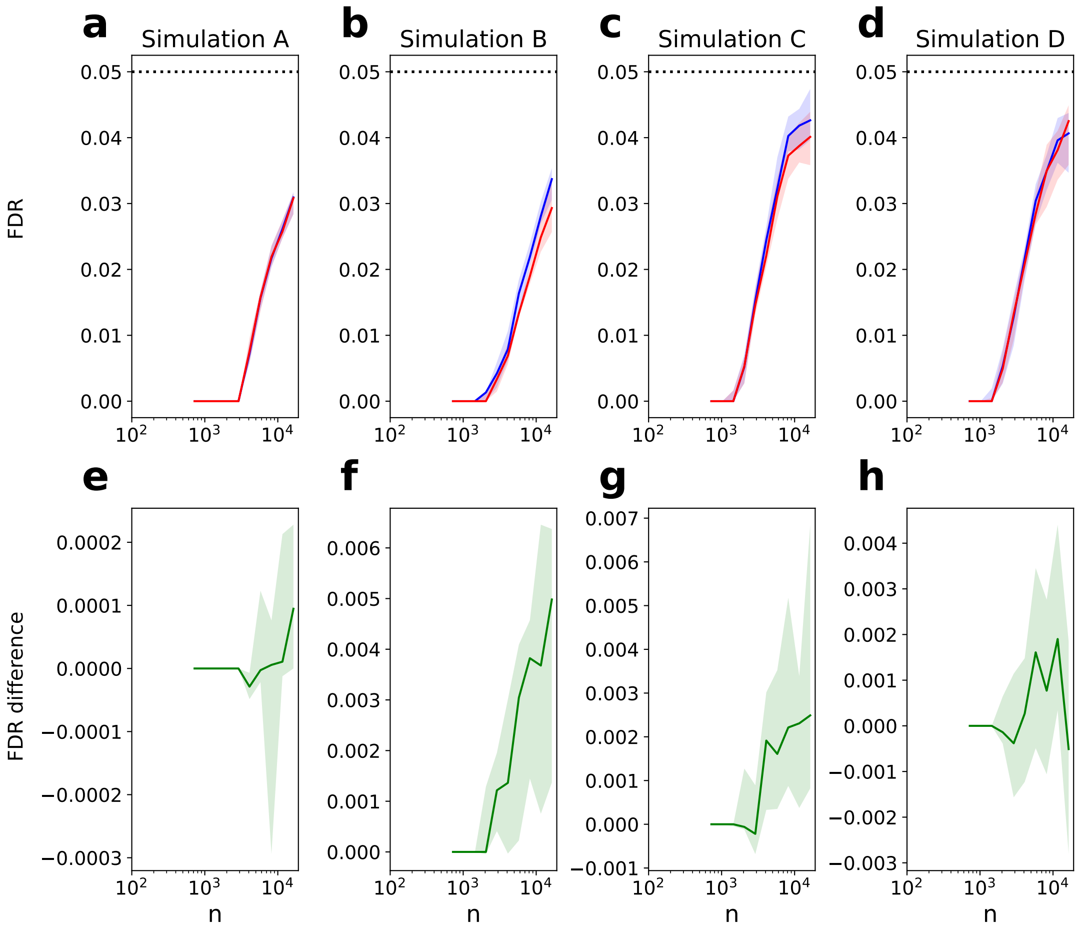

Figure S11: As Figure S9a to d and S10a to d but now using the theoretical value $\kappa = n - 1 - (p - 2)$ to estimate significance levels.

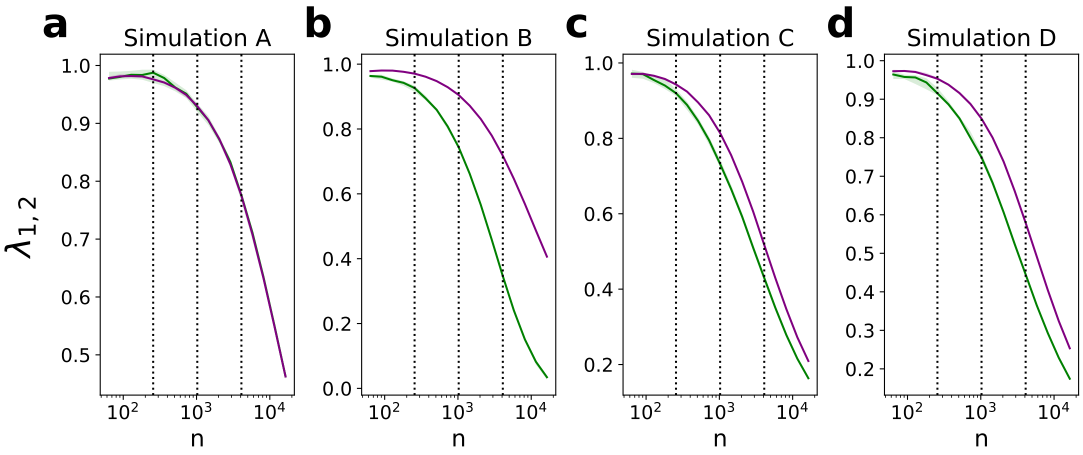

Figure S12: Regularization parameters λ_1 (green) and λ_2 (purple) for simulation studies A to D for different sample sizes. The vertical, dotted lines correspond to $n = 256$, $n = 1,024$, and $n = 4,096$. The respective pairs (λ_1, λ_2) were evaluated to verify that p-value distributions are flat under the null hypothesis ($H_0 : \rho = 0$).

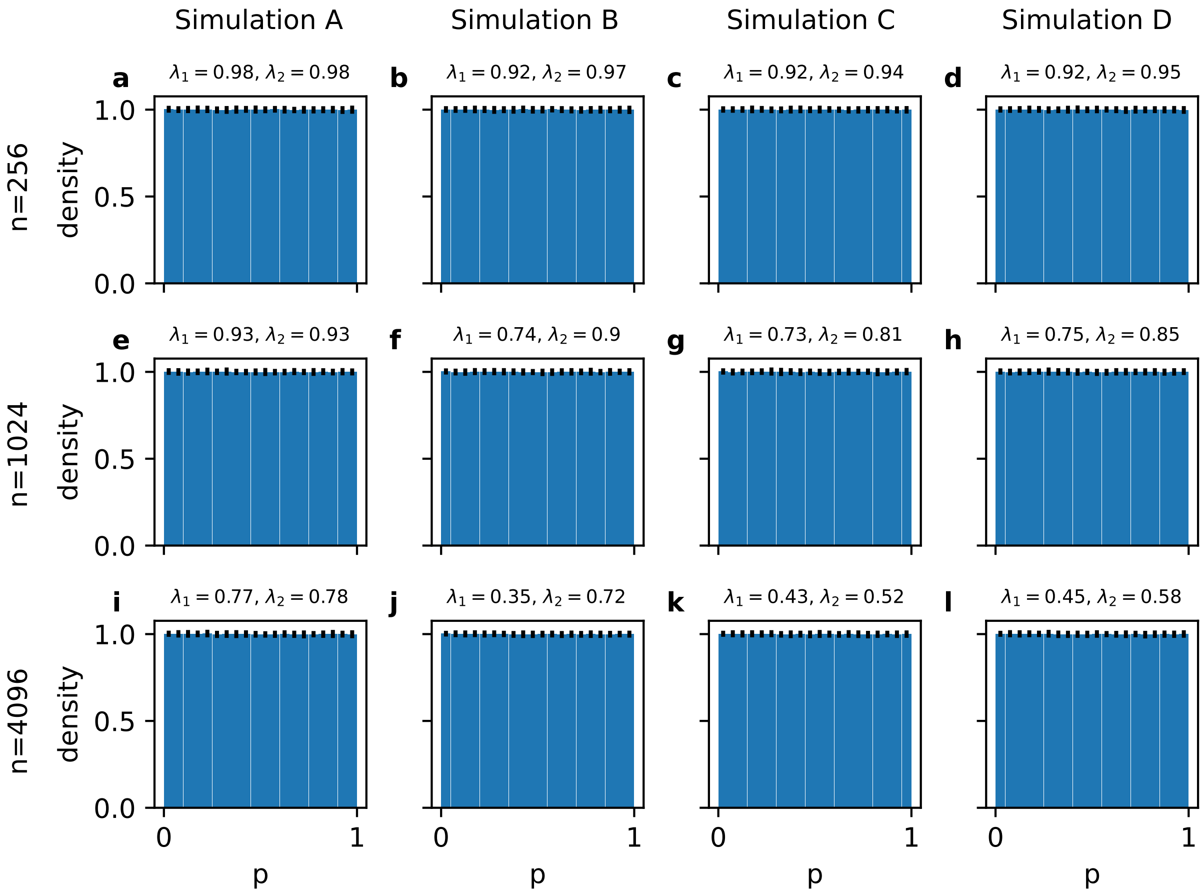

Figure S13: Distributions of p-values estimated by DRAGON for data simulated under the null hypothesis ($H_0 : \rho = 0$) for given pairs of the regularization parameters λ_1 and λ_2. The (λ_1, λ_2)-pairs are given at the top of Figures a to l and were derived as described in the main article, cf. also Figure S12. Their corresponding sample size n and simulation study are given at the left side and at the top, respectively. Error bars represent ± 1 standard deviation across 10 simulation runs.

4. TCGA analysis dataset

The dataset consists of one primary tumor sample from each of 765 women. The distribution of subtype, race, and ethnicity are shown below in Table S1.

mRNA subtype		
	Basal	132
	Her2	46
	Luminal A	412
	Luminal B	141
	Normal	34
Race		
	American Indian or Alaska Native	1
	Asian	38
	Black or African American	157
	White	554
	Not reported	15
Ethnicity		
	Hispanic or Latino	36
	Not Hispanic or Latino	661
	Not reported	68

Table S1: Basic characteristics of the TCGA dataset used in the breast cancer example.

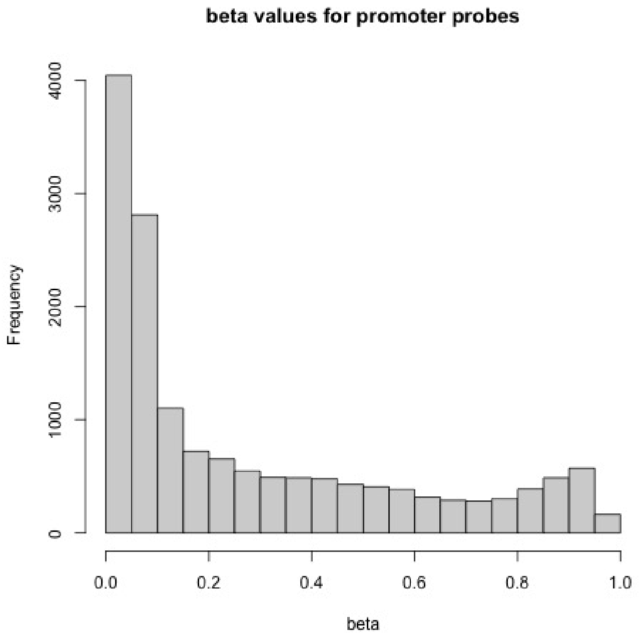

Figure S14: Distribution of mean promoter methylation across TFs in our analysis.

5. Preprocessing of TCGA methylation data

Mapping array probes to TF promoters

The TCGA methylation files consist of one beta value per probe per sample. In this application, we consider promoter methylation of transcription factors (TFs) only. To construct one methylation value per TF per sample, we first filtered probes to only those probes within 1500 base pairs upstream of the transcription start site (TSS) for each TF. Next, probes meeting this criterion for more than one TF were mapped to the closest downstream TF. Finally, the beta values for each probe in the promoter region for each TF were averaged within each sample, yielding one methylation value per TF per sample.

Distribution of promoter methylation values

The distribution of these methylation values for each of the 1590 TFs in our dataset is shown in Supplementary Figure S14. Rather than the typical bimodal distribution expected in methylation data, these methylation values tend towards a right-skewed, unimodal distribution. This may be due to the fact that all samples are from cancerous tissue and promoter hypomethylation (particularly in CpG islands) is characteristic of cancers [3]. This hypothesis is supported by the distribution of the individual probes across CpG island labels (island, shelves, and shores; Supplementary Figure S15).

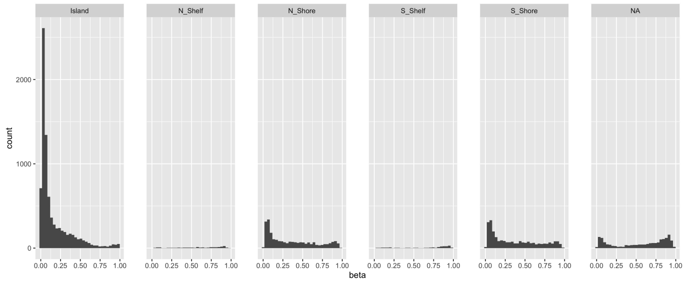

Figure S15: The distribution of methylation beta values shows an emphasis on probes in CpG islands and a general trend of hypomethylation in probes annotated to islands, shelves, and shores vs. probes without a CpG island-related annotation ('NA').

Missing data and data transformation

Genes missing beta values for greater than 20 percent of the samples were removed from the analysis (N=33). For genes missing from ≤ 20 % of the samples, missing promoter beta values were imputed with mean imputation. Prior to input into DRAGON, promoter methylation was transformed to approximate Gaussianity using the nonparanormal transformation in the R package `huge` [4, 5].

6. Preprocessing of TCGA RNA-seq data

No measures of TPM were missing in the gene expression data; however, many counts had a value of 0 or 1 TPM. We included only TFs that were expressed with TPM > 1 in at least 20 percent of the data. This criterion omitted 318 of the TFs. TPMs were transformed to an approximately Gaussian distribution using a log transformation. To handle the cases where TPM=0, we set the transformed TPM equal to log(TPM+1).

7. Summary of DRAGON model

We ran DRAGON on 765 samples of 1311 gene expression measurements and 1557 gene-level methylation measurements after preprocessing as described above. Recall that DRAGON does not require measurements on the same variables across omics layers; here, both expression and methylation levels were available for 1297 genes.

The optimal tuning parameters were determined to be $\lambda_1 = 0.058$ and $\lambda_2 = 0.031$, where λ_1 governs the shrinkage of the methylation data and λ_2 governs the shrinkage of the gene expression data. The estimated parameters of the probability distribution in Equation (10) of the main manuscript were $\kappa_{11} = 12725.61$, $\kappa_{22} = 5175.30$, and $\kappa_{12} = 815.94$.

8. Community detection methodology

Community detection was performed using the `cluster_fast_greedy` algorithm as implemented in the `igraph` R package[6, 7]. In this algorithm, the weight of an edge in the graph represents the strength of

the connection between two nodes. Because the DRAGON network contains both positive and negative edge weights, edge weights were transformed to their absolute value prior to community detection. This transformation reflects the paradigm that we are interested in communities of nodes that are conditionally associated, not whether the association is positive or negative.

To conduct the over-representation analysis, the `fora` function of the `fgsea` R package was used[8]. Reactome gene sets with at least 3 genes were considered (`minSize` = 3). Importantly, a single TF may be represented by two separate nodes in the DRAGON network, one representing its methylation and one representing its expression. Therefore, the universe of possible genes considered for the ORA has twice the size of the number of TFs included in the DRAGON model and consists of two entries for each TF: one corresponding to its methylation and one corresponding to its expression. We additionally considered an ORA assessing enrichment for methylation only and one assessing enrichment for expression only. A Reactome pathway was considered to be over-represented in a community if its FDR was less than 0.05 in at least one of these three methods (Benjamini-Hochberg FDR as implemented in `fgsea`).

9. BRCA hub nodes

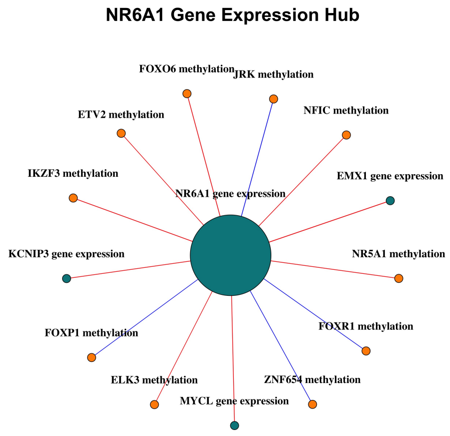

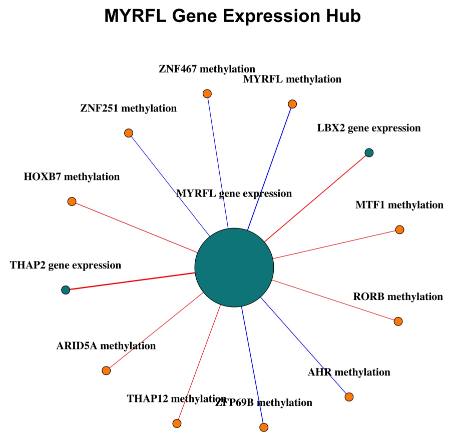

Figure S16: Neighborhoods of NR6A1 and MYRFL in the DRAGON breast cancer network. Turquoise nodes represent gene expression and orange nodes represent promoter methylation. Larger nodes have higher node degrees. Edges with FDR < 0.05 are shown.

10. Community analysis

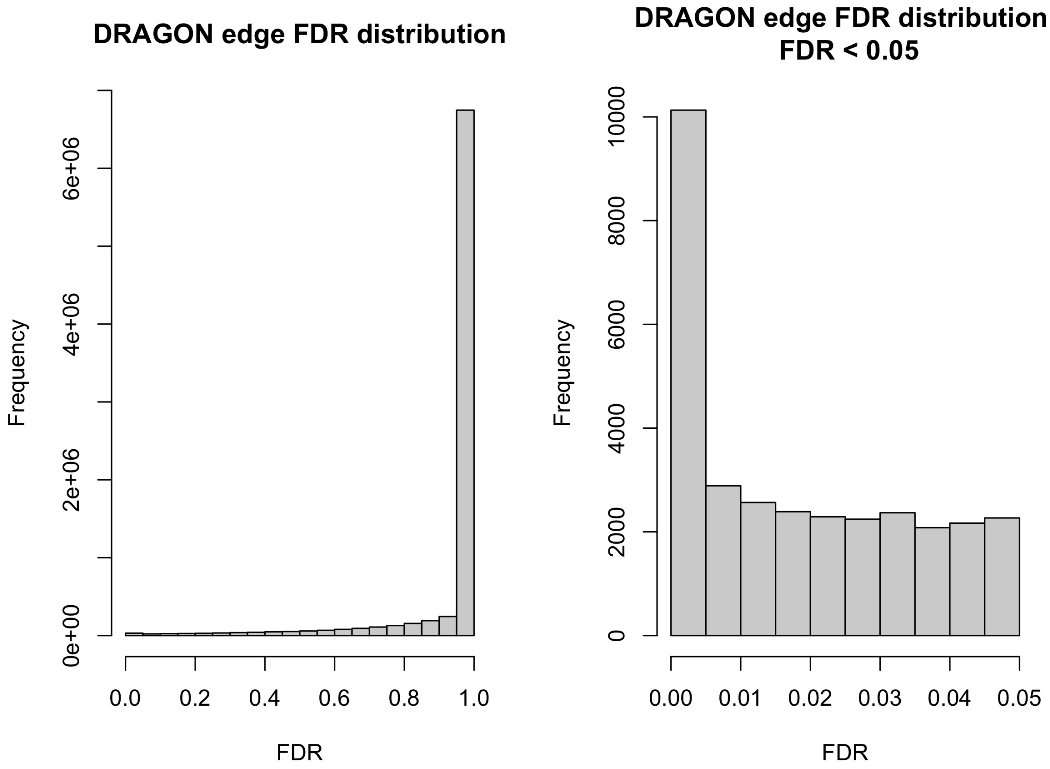

Figure S17: Distribution of Benjamini-Hochberg adjusted p-values (FDR) for the DRAGON BRCA network. Based on the shape of this distribution, edges with $FDR < 0.005$ were considered true edges and carried forward in the analysis.

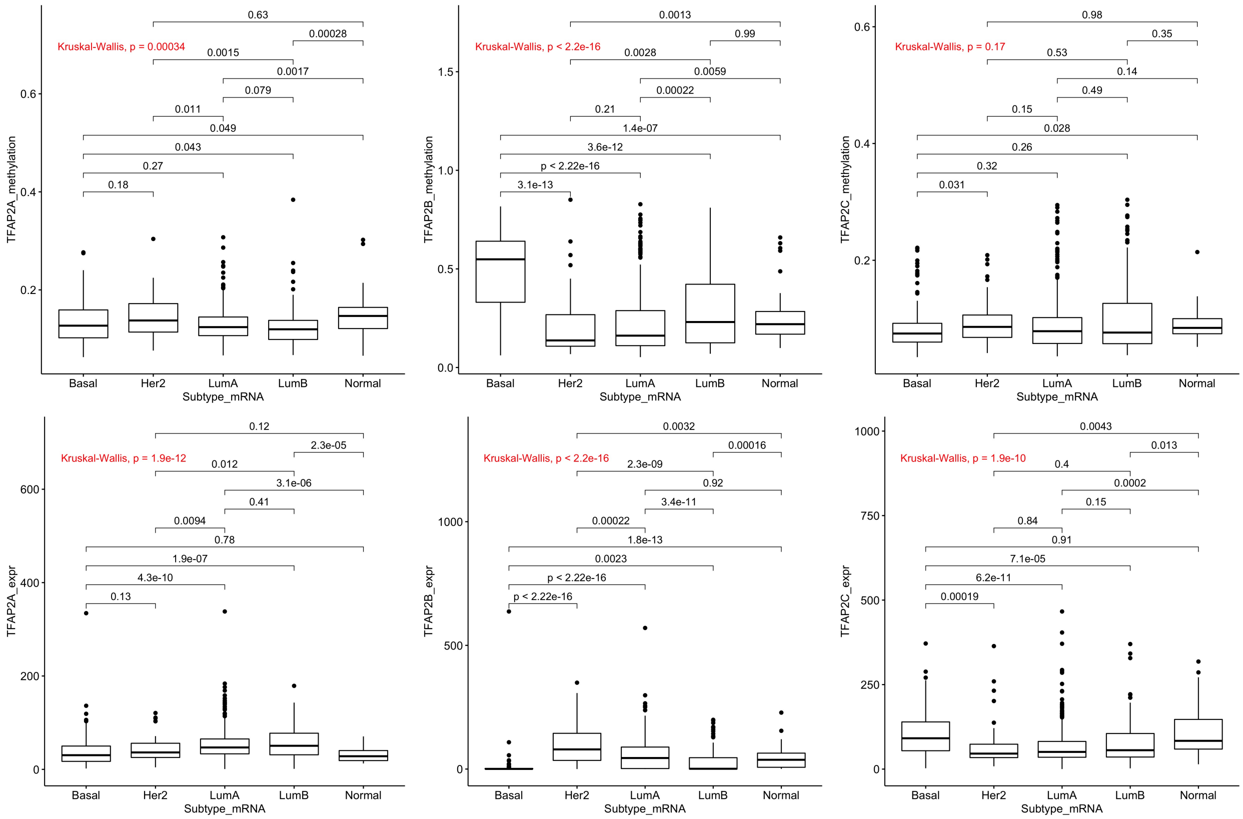

Figure S18: Subtype-specific methylation and expression of TFAP2 family transcription factors, which are key players in the enrichment analysis for Community 5.

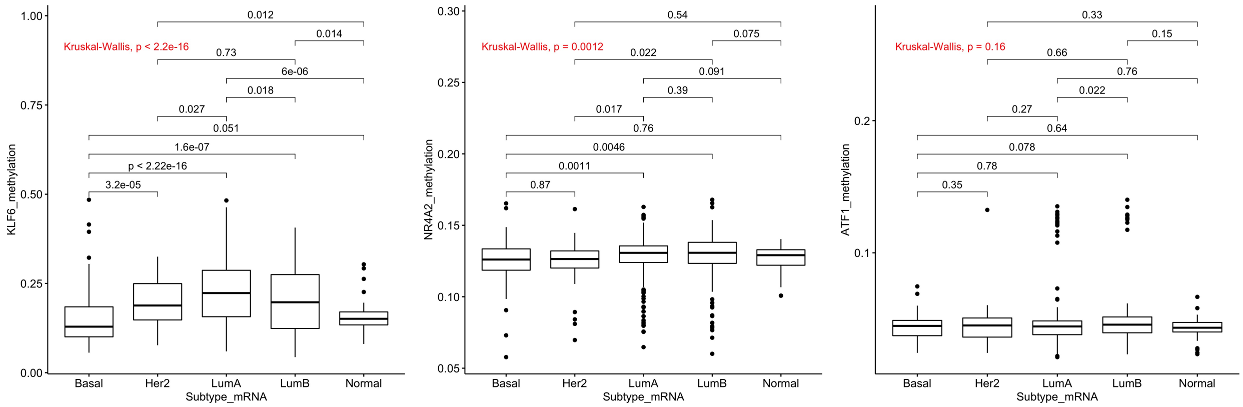

Figure S19: Subtype-specific methylation of key methylation nodes in Community 38 (KLF6 and NR4A2), vs. subtype-specific methylation of a housekeeping gene (ATF1).

References

[1] R Core Team. *R: A Language and Environment for Statistical Computing*. R Foundation for Statistical Computing, Vienna, Austria, 2021.

[2] Seongho Kim. *ppcor: Partial and Semi-Partial (Part) Correlation*, 2015. R package version 1.1.

[3] Emmanouil Bouras, Meropi Karakioulaki, Konstantinos I Bougioukas, Michalis Aivaliotis, Georgios Tzimagiorgis, and Michael Chourdakis. Gene promoter methylation and cancer: An umbrella review. *Gene*, 710:333–340, 2019.

[4] Han Liu, John Lafferty, and Larry Wasserman. The nonparanormal: Semiparametric estimation of high dimensional undirected graphs. *Journal of Machine Learning Research*, 10(Oct):2295–2328, 2009.

[5] Tuo Zhao, Han Liu, Kathryn Roeder, John Lafferty, and Larry Wasserman. The huge package for high-dimensional undirected graph estimation in r. *The Journal of Machine Learning Research*, 13:1059–1062, 2012.

[6] Pascal Pons and Matthieu Latapy. Computing communities in large networks using random walks. In *International symposium on computer and information sciences*, pages 284–293. Springer, 2005.

[7] Gabor Csardi, Tamas Nepusz, et al. The igraph software package for complex network research. *InterJournal, complex systems*, 1695(5):1–9, 2006.

[8] Gennady Korotkevich, Vladimir Sukhov, Nikolay Budin, Boris Shpak, Maxim N Artyomov, and Alexey Sergushichev. Fast gene set enrichment analysis. *BioRxiv*, page 060012, 2021.
